# Tuning Molecular Transformations and Accumulation of Reactive Species in Plasma-Treated Liquids via Organic Biomolecules

**DOI:** 10.1021/acsomega.6c06500

**Published:** 2026-10-27

**Authors:** Valeria Veronico, Michele Casiello, Loris Grossi, Roberto Gristina, Vincenza Armenise, Pietro Favia, Eloisa Sardella

**Affiliations:** † 9295 Universita degli Studi di Bari Aldo Moro, Dipartimento di Chimica, via Orabona 4, Bari 70125, Italy; ‡ 9327 Istituto di Chimica dei Composti Organo Metallici, Consiglio Nazionale delle Ricerche Sezione di Bari, c/o Universita degli Studi di Bari Aldo Moro Dipartimento di Chimica, Via Orabona 4, Bari I-70126, Italy; § Universita degli Studi di Bologna, Dipartimento di Scienze per la Qualita della Vita, Corso d’Augusto, 237, Rimini I-47921, Italy; ∥ 518830 Istituto di Nanotecnologia Consiglio Nazionale delle Ricerche Sede di Bari, c/o Universita’ degli Studi di Bari Aldo Moro Dipartimento di Chimica, Via Orabona 4, Bari 70126, Italy

## Abstract

Plasma processing of liquids is a sustainable platform for green chemistry and biomedical applications, enabling the synthesis of inorganic and organic molecules without harmful reagents and with compatibility with renewable electricity. PTWS constitute reservoirs of reactive oxygen and nitrogen species, and their composition is governed not only by the experimental conditions of the plasma employed for their generation, but also, and predominantly, by the chemical composition of the precursor liquid. In this study, we demonstrate that organic solutes actively modulate RONS chemistry. Phenolic compounds such as l-tyrosine and resorcinol enhance H_2_O_2_ production while differently affecting nitrogen species, leading to either scavenging or overproduction of NO_2_^–^. Mass spectrometry reveals a strong correlation between molecular structure, plasma-induced modifications, and resulting RONS dynamics. These findings redefine PTWS as dynamic, reactive environments rather than passive reservoirs by also explaining the prolonged activity of such liquids. Beyond plasma medicine, they provide a framework for optimizing RONS-based therapies and contribute to the broader understanding of nonequilibrium chemistry in complex liquids. The results also open opportunities in water treatment and sustainable chemical transformations, positioning PTWS as versatile tools for controlling redox processes in complex aqueous systems.

## Graphical abstract

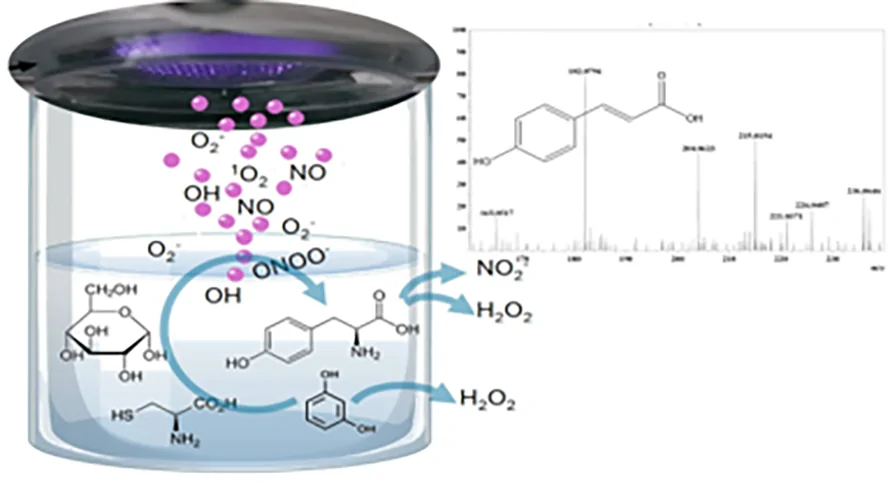

## 1 Introduction

Cold Atmospheric Plasmas (CAPs) are partially ionized gases, quasi-neutral, composed by the feed-gas species and their fragments (i.e. electrons, ions, atoms, and radicals), operating in nonequilibrium conditions at atmospheric pressure.^1^ In recent years, CAP research has rapidly evolved from fundamental plasma physics toward high-impact biomedical applications, establishing the field of plasma medicine^2–4^ Within this framework, CAPs are recognized as powerful sources of reactive oxygen and nitrogen species (RONS), including H_2_O_2_, O_3_, OH, O_2_^–^, O, ONOO^–^, NO, NO_3_^–^ and NO_2_^–^,^5,6^ whose controlled delivery has shown strong potential in wound healing,^7,8^ antimicrobial treatments^9^ and oncology.^10,11^

Beyond direct plasma exposure, a rapidly expanding and increasingly translational approach is the use of plasma-treated water solutions (PTWS), in which plasma-generated species are transferred into liquids to create chemically active, long-lived formulations.^12,13^ In this sense, plasma can be viewed as a unique on-demand chemical reactor, capable of generating a dynamic and tunable “reactive cocktail” whose composition is governed by the interplay between gas-phase chemistry and liquid-phase reactivity. In air (or a mixture of N_2_ and O_2_) environments, a broad spectrum of RONS is typically formed (i.e. O_3_, O, ^1^O_2_, ·NO_*x*_, N_2_*, N, and ONOO^–^), along with solvated electrons (e_solv_), whereas, in the absence of nitrogen in a humid environment, the plasma chemistry is mainly driven by reactive oxygen species (i.e. ·OH, H_2_O_2_, and ·OOH).^14^ In both cases, short-lived primary species in the plasma phase trigger cascades of secondary reactions in the plasma-liquid interface that leads to the accumulation of longer-lived oxidants and nitrogen species such as H_2_O_2_, NO_2_^–^, and NO_3_^–^, which ultimately define the biological and chemical signature of PTWS.^15,16^

Despite significant progress in modeling and characterization, most mechanistic studies have been performed in simplified aqueous systems,^14,17,18^ with implicit assumptions that often overlook the complexity of real biological environments. However, clinically relevant liquids such as cell culture media,^19,20^ saline solutions,^21^ Phosphate-Buffered Saline (PBS),^22^ and Ringer’s lactate^23,24^ are chemically rich matrices which often contain amino acids, vitamins, metabolites, and electrolytes that are far from passive. Increasing evidence now shows that these solutes actively reshape plasma chemistry through scavenging, redox cycling, and the formation of secondary reaction products, challenging the long-standing view of PTWS as mere carriers of plasma-generated species.^25,26^ Previous findings further demonstrated that RONS yields and speciation are strongly dependent on the composition of the starting solution, highlighting the need to move beyond water-centric models *(27).* Emerging studies also suggest that organic solutes may critically contribute to PTWS aging and to the in situ generation of secondary reactive species, opening new and still largely unexplored chemical pathways.^28,29^

In this context, a more realistic and mechanistically grounded understanding of plasma–biomolecule interactions is becoming a key frontier in plasma medicine. Here, we address this challenge by investigating PTWS generated from aqueous solutions of five representative biorelevant organic molecules, namely l-tyrosine, l-cysteine, nicotinamide, d-glucose, and resorcinol, selected as model components of biological fluids and clinical formulations. These compounds span key functional motifs found in living systems: redox-active thiols, phenolic structures, metabolic cofactors, reducing sugars, and phenolic antioxidants.^29–32^

An important aspect of liquid functionalization via plasma lies in the treatment of solutions already used in clinical settings, which may be supplemented with pro-oxidant organic molecules such as tyrosine.^33^ In these complex buffered systems, containing salts and low-molecular-weight organics, plasma exposure triggers a dense network of coupled reactions leading to both depletion and ex novo formation of reactive species. RONS production and molecular transformations were quantified in this research using colorimetric assays for H_2_O_2_ and NO_2_^–^, combined with high-resolution mass spectrometry to resolve plasma-induced chemical fingerprints. In plasma-liquid interactions, reactive species originate either within the plasma phase^34^ or through interactions with the surrounding atmosphere.^35^ The resulting RONS “cocktail” includes a complex array of short- and long-lived species (O, O_3_, ^1^O_2_, •OH, O_2_^•–^/•OOH, •NO, ONOO^–^ and OONOO^–^, H_2_O_2_, NO_2_^–^, NO_3_^–^).^27^ When target organic molecules are present the chemical environment becomes even more intricate.

In such a highly reactive milieu, quantifying short-lived organic and inorganic radicals using direct Electron Paramagnetic Resonance (EPR) spin trapping presents major analytical challenges. Specifically, competitive side reactions and the limited selectivity of common spin traps can easily introduce significant artifacts.^14^ Furthermore, given the rapid evolution of the system toward a steady state of stable products (H_2_O_2_, NO_2_^–^, etc.) and the difficulties of EPR in these complex matrices, direct spin trapping of specific organic radicals was deliberately avoided to ensure data reliability.

Instead, our investigation intentionally focuses on spectrophotometric quantification of stable H_2_O_2_, NO_2_^–^ as markers of RONS produced in the liquids; within complex matrices containing organic substrates, transient short-lived radicals undergo ultrafast reactions with the organic precursors, leaving behind highly specific, stable chemical “footprints” (e.g., hydroxylated derivatives, dimers, and nitration products).^27^ Liquid Chromatography–Mass Spectrometry (LC-MS) serves as an exceptionally robust and sensitive analytical platform to intercept these terminal end points, enabling the accurate back-extrapolation and elucidation of the underlying radical-driven reaction mechanisms. Thus, in this paper, short-lived species (OH, O_2_^–^, ONOO^–^) are proposed as key transient intermediates based on established plasma chemistry and the detected stable footprints,^12,27^ rather than directly quantified transients. Although plasma-treated aqueous solutions have been extensively studied, organic solutes are predominantly regarded as passive target molecules or RONS scavengers. Here, we demonstrate a fundamental paradigm shift: specific organic biomolecules act as active chemical modulators that dictate the accumulation pathways of liquid-phase reactive species, actively generating secondary ROS/RNS rather than merely consuming primary RONS.

By uncovering how organic solutes interact with primary plasma species to produce distinct secondary intermediates, this work moves away from treating PTWS as a static chemical cocktail toward a framework of rational, targeted design. Leveraging specific biomolecular additives allows for the precise tuning of reactive species profiles—a capability critical for tailoring plasma-treated liquids to biomedical and therapeutic applications where selective RONS control dictates clinical efficacy and safety.

Beyond therapeutic translation, this predictive understanding of emergent plasma–biomolecule chemistry provides a versatile foundation for optimizing plasma–liquid processes in environmental remediation, wastewater management, and green synthesis.

## 2 Materials and Methods

### 2.1 PTWS Generation in a Planar Dielectric Barrier Discharge

To generate PTWS of each organic molecule, we used a planar Dielectric Barrier Discharge (DBD) source. The reactor (Figure 1a) consists of a discharge unit and a Teflon flow unit, the latter equipped with gas connections and with a dry diaphragm pump (Pfeiffer Vacuum; Aßlar). The operative pressure, measured with an MKS Baratron, was kept constant at 760 Torr. The discharge unit consists of a stainless-steel grounded grid positioned 4 mm far from a copper disc high-voltage (HV) electrode (7.6 cm dia., 0.5 mm thick), covered with a 1 mm thick glass dielectric disc. The HV copper electrode is connected to a microsecond-pulsed generator (Alma-Pulse; AlmaPlasma s.r.l.). For the experiments here reported, we generated the plasma in a region as wide as the HV electrode, by applying a 6–9 kHz electric field at 10–13.5 kV of peak-to-peak voltage (Vpp), pulsed with a 25% duty cycle (DC; 25 ms plasma on, t_on_) over a period (t_on_ + t_off_) of 100 ms. This electrode arrangement allowed the generation of filamentary discharges stable within the range of the used parameters frequency, voltage, treatment time, and gas mixture.

**Figure 1:**
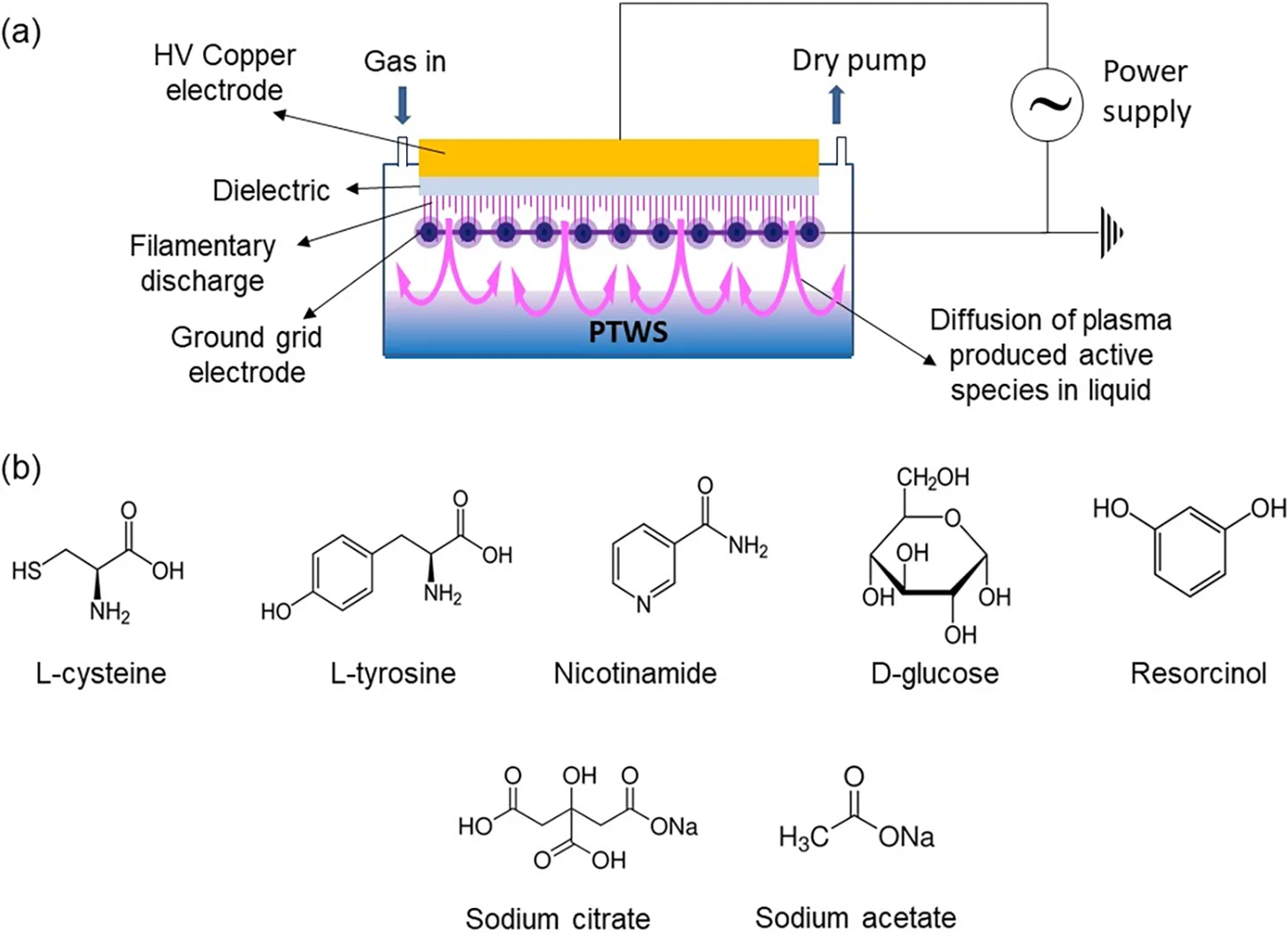
(a) Schematic representation of the DBD source used for PTWS generation; (b) chemical structures of the organic molecules included in the water solutions tested in this research.

Once the DBD is ignited, the treatment is achieved by the diffusion of RONS from the plasma to the liquid through the ground grid (pink arrows in Figure 1a). The DBD is housed in a glass chamber (150 mm diameter), filled with liquid to be treated. In each experiment, up to 30 mL of each water solution was placed 3 mm below the grid electrode inside a closed chamber to minimize ambient air ingress into the plasma zone. The liquid-discharge headspace was purged with the gas feed for 1 min before igniting the plasma. The selection of treatment durations (45 s up to 5 min) was set after our previous optimization studies;^27^ they represent an optimal operational window that yields sufficient RONS concentrations to induce the target chemical/biological processes, while preventing overoxidation, thermal degradation, or sample evaporation artifacts observed at prolonged exposure times (e.g., >10 min). Pure O_2_ (Air Liquid, 99.999%) or synthetic air (Air Liquid, 99.999%) was fed at a constant flow rate 0.5 L·min^–1^–1 L·min^–1^, regulated with MKS mass flow controllers.

PTWS were generated from double-distilled water (dd-H_2_O) and water solution of l-cysteine, l-tyrosine, nicotinamide, resorcinol, and d-glucose (Figure 1b). The solutions were prepared by dissolving pure cysteine (cat. No. C8755, Sigma-Aldrich), tyrosine (cat. No. T-3754, Sigma-Aldrich), nicotinamide (cat. No. N3376, Sigma-Aldrich), or resorcinol (cat. No. 38047, Sigma-Aldrich) powders in dd-water. For l-cysteine and l-tyrosine complete dissolution was achieved in ultrasonic bath for 3 h at 30 °C. d-glucose solutions were prepared by dilution from a 5% glucose water solution (Eurospital S.P.A.). To better elucidate the effect of organic molecules on RONS generation, we tested increasing concentrations of the molecules in the range of 0–0.38 mM for l-cysteine, 0–1.66 mM for l-tyrosine, 0–24.60 mM for nicotinamide, 0–27.2 mM for resorcinol, and 0–305.06 mM for d-glucose. Additionally, an Electrolyte Rehydration III Solution (SIII; Fresenius Kabi), Food and Drug Administration (FDA)-approved isotonic saline formulation intended for intravenous infusion, was employed as a clinically relevant model system to investigate plasma-induced modifications in solution chemistry. SIII consists of an aqueous mixture of inorganic chlorides and low-molecular-weight organic salts, including NaCl (85.60 mM), KCl (10.06 mM), CaCl_2_ (3.15 mM), MgCl_2_·6H_2_O (1.52 mM), sodium acetate (78.02 mmol·L^-1^), and sodium citrate (2.90 mM). SIII was chosen as the most appropriate medium for this study due to its pH-buffering action, which relies on acetate and citrate instead of lactate that sometimes requires external pH adjustment after plasma treatment.^36^

### 2.2 Colorimetric Detection of H_2_O_2_ and NO_2_^–^

RONS generation in PTWS was monitored by analyzing the amount of H_2_O_2_ and NO_2_^–^ produced in the treated solutions. The Griess assay was used for quantitative analysis of NO_2_^–^ ions: 13 mg of reagent powder (cat. no. 1.14776.001, Merck Millipore) were added to 1 mL of liquid sample and left to react 10 min before measuring the absorbance at 525 nm. A homemade copper-neocuproine assay was used to detect H_2_O_2_ by sequentially adding 100 μL of a 0.012 M water solution of CuSO_4_ (prepared from cat. no. 31293, Sigma-Aldrich), 100 μL of phosphate buffer 0.01 M at pH = 7 (prepared from Na_2_HPO_4_ cat. no. 30427, Riedel-de Haën, and NaH_2_PO_4_·H_2_O cat no. 480087, Carlo Erba), and 100 μL of neocuproine (cat. no. 1501, Sigma-Aldrich) 0.06 M in methanol to 700 μL of sample. The reaction was allowed for 10 min before measuring the absorbance at 455 nm. UV–vis absorbance measurements were performed with a Cary 60 UV–vis spectrophotometer (Agilent, Santa Clara, CA, USA) in the spectral range 200–800 nm with a 5 nm resolution. Disposable plastic cuvettes (cat. no. 223-9955, Bio-Rad, Hercules, CA, USA) with a semimicro volume of 1 mL and a 10 mm optical length were used. The assays were specifically validated in case of PTWS.^37^ The data were computed with the GraphPad Prism software version 6.1 to elaborate statistical results of measurements, shown as the mean value ± Standard Deviation (SD). A one-way ANOVA analysis was performed with a subsequent Tukey’s Multiple Comparison Test by assuming a normal distribution in the data. All p-values <0.001 were considered statistically significant.

### 2.3 High-Resolution Mass Spectrometry

Mass spectra were obtained in the electrospray ionization (ESI) positive- and negative-ion modes with an LC-MS-IT-TOF (Shimadzu) mass spectrometer equipped with a binary pump (NexeraXR, LC-20ADxr), an autosampling system (NexeraXR, SIL-20ADxr), and a photodiode array (SPD-M20A) detector. The curved desolvation line (CDL) and heat block temperature were set at 250 °C, mass screening range at 100–500 *m*/*z*, detector voltage at 1.7 kV, and the nebulizing gas (i.e., N_2_) flow at 1.5 L·min^-1^.

A volume of 2 μL of untreated or PTWS of the organic molecules was directly infused in the spectrometer by using an acid mobile phase (i.e., a mixture 4-fold v/v of methanol and 3-fold v/v of acetonitrile and 0.1% v/v of formic acid) to improve the detection sensitivity. The solutions were electro sprayed at a flow rate of 0.3 mL·min^–1^. The qualitative analysis was carried out by comparing the exact masses with the isotopic distributions and through a study of the fragmentation pattern of MS^2^ when available. The LCMS solution (V3.80.410, Shimadzu) software was used for data acquisition and processing; the qualitative characterization was processed with the Shimadzu LabSolutions Lite V5.82 software package (Formula Predict and Accurate Mass Calculator).

## 3 Results and Discussion

### 3.1 Effect of Organic Molecule Concentration on H_2_O_2_ and NO_2_^–^ Generation

The results of H_2_O_2_ and NO_2_^–^ detection in PTWS generated from pure water, l-cysteine, l-tyrosine, nicotinamide, resorcinol, and d-glucose solutions are reported in Figures 2 and in [Fig fig3], respectively. As reported in our previous paper,^27^ the formation of H_2_O_2_ resulted completely inhibited after the treatment in pure water (the data point in the *x* axis corresponds to 0 in each graph of Figure 2) and in its solutions of l-cysteine (Figure 2a), nicotinamide (Figure 2c), and d-glucose (Figure 2d), regardless of the feed used and at any concentration of the tested molecules. On the contrary, considerable amounts of H_2_O_2_ (up to hundreds of μM) were generated in the presence of l-tyrosine (Figure 2b) and resorcinol (Figure 2e). In both cases, the H_2_O_2_ amount increased as a function of the concentration of resorcinol and l-tyrosine and shifted toward higher values when the DBDs were ignited in pure O_2_ in comparison to the air feed.

**Figure 2:**
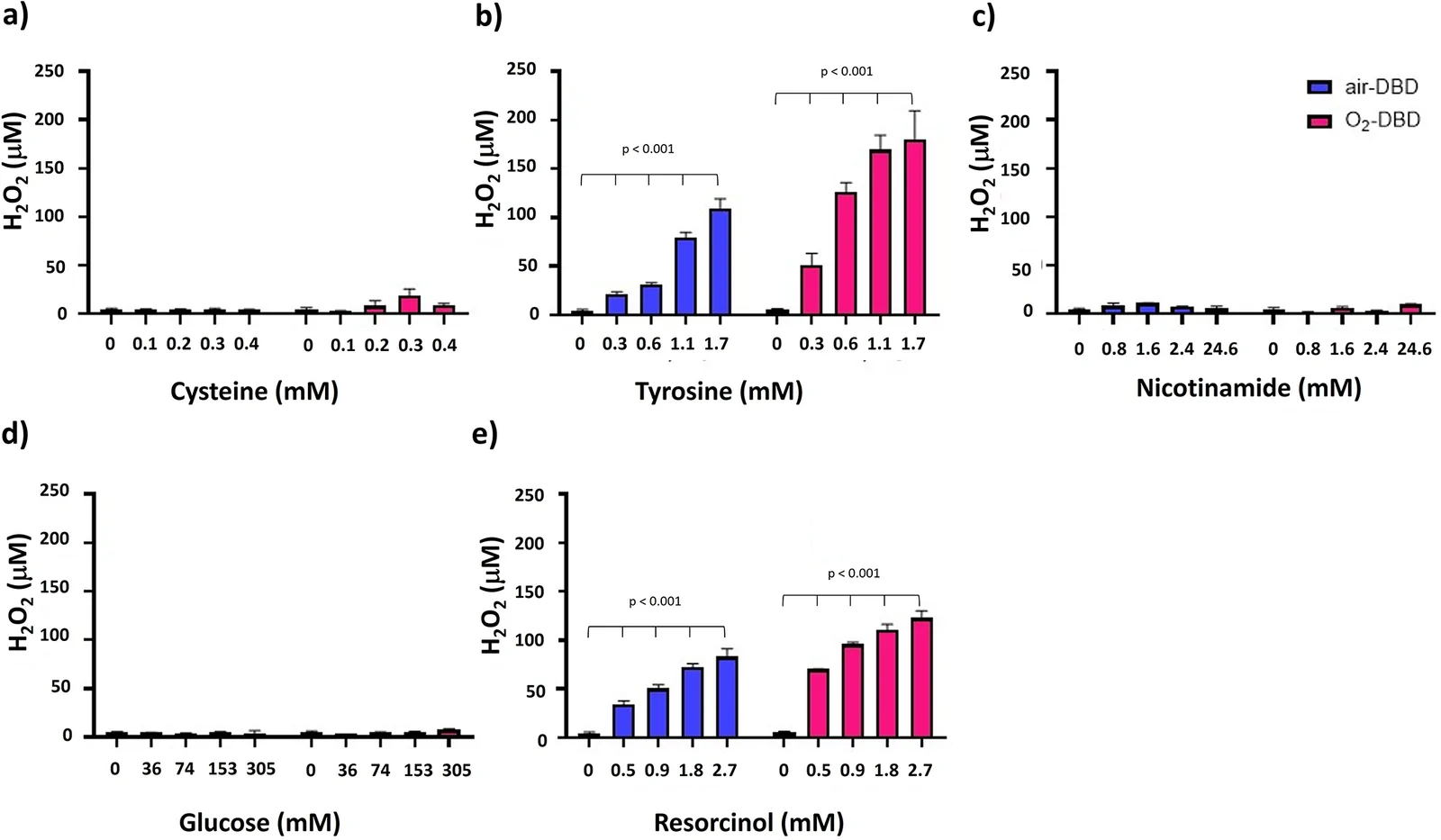
H_2_O_2_ concentration in PTWS generated in 2 mL of water solutions with increasing concentrations of (a) l-cysteine (0–0.4 mM); (b) l-tyrosine (0–1.7 mM); (c) nicotinamide (0–24 mM); (d) d-glucose (0–305 mM); (e) resorcinol (0–2.72 mM) with 3 min CAP treatment (13.5 kV, 6 kHz, 25% DC, 0.5 L/min) in air or O_2_ feed. The data points at 0 in the *x* axis correspond to treatments in dd-water.

**Figure 3:**
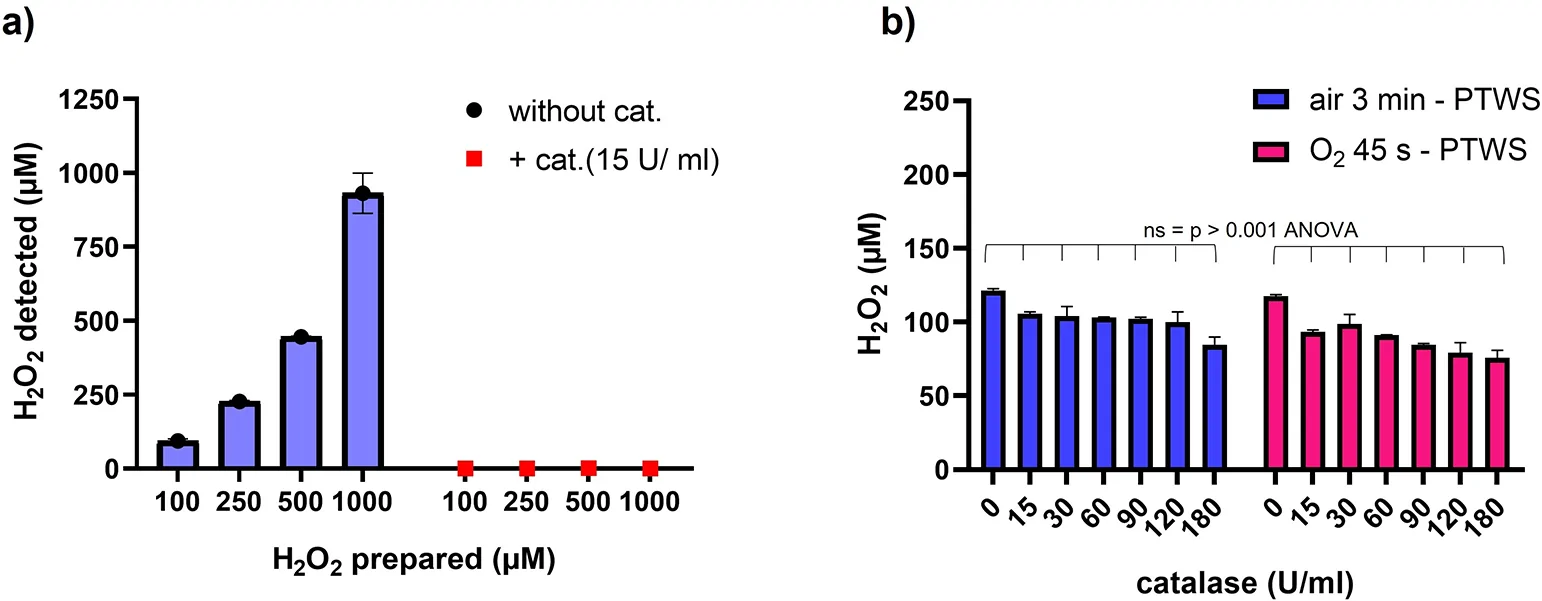
Quantification and enzymatic degradation of H_2_O_2_. (a) Calibration curve of detected H_2_O_2_ concentrations in dd-H_2_O in the absence (black dots/blue bars) and presence (red squares) of catalase (15 U/mL), demonstrating complete scavenger specificity. (b) Effect of increasing catalase concentrations (0–180 U/mL) on H_2_O_2_ levels in plasma-treated SIII generated using air (3 min, blue bars) or O_2_ (45 s, red bars). Data represent mean ± SD. Statistical analysis was performed using one-way ANOVA (ns = *p* > 0.001).

H_2_O_2_ could not be produced in dd water or in PBS. It is our opinion that this is due to the negligible evaporation in a planar DBD with a closed system that limits the generation of free hydroxyl radicals (OH) involved in H_2_O_2_ generation.^14,27^ Typically, OH radicals are formed in the plasma phase from dissociation of water molecules from humid gas feeds or from water evaporation,^27^ or they can be directly formed in water due to reactions with other RONS or UV photolysis.^27^ In the case of our planar DBD source, these pathways for OH generation can be neglected since the discharge is ignited under anhydrous conditions (no humidity in the feed) and it is switched on the liquid with no contact.

Therefore, it can be supposed that the generation of H_2_O_2_ in l-tyrosine and resorcinol solutions could proceed through reaction pathways alternative to OH recombination. Our hypothesis is that under our conditions, these pathways rather include the formation of radicals on specific sites of the organic molecules. l-tyrosine and resorcinol (Figure 1b) share a phenolic structure, which we assumed to be targeted by plasma-diffused ROS to generate H_2_O_2_ into the liquid through derivatives of such organic molecules. According to this hypothesis: (i) H_2_O_2_ was generated only in the presence of l-tyrosine and resorcinol, while it was not generated in the presence of l-cysteine, nicotinamide, and d-glucose, because no phenol groups are present in their structures; (ii) the treatment of l-tyrosine and resorcinol solutions with O_2_-DBDs, where anhydrous ROS are abundantly generated, produced greater amounts of H_2_O_2_ in PTWS in comparison to air-fed DBDs, where ROS could be less abundant.

Similar results were obtained in our published research, in which a linear correlation of H_2_O_2_ produced with the concentration of tyrosine added was observed in clinically relevant solutions like SIII, while no H_2_O_2_ production was observed in pristine SIII solutions without tyrosine.^33^ For the present paper, in order to verify if H_2_O_2_ is produced from reactions mediated by organic components in the liquid, we added catalase to an SIII supplemented with tyrosine (1.7 mM) O_2_-plasma-treated for 45 s and air plasma-treated for 3 min characterized by the same concentration of H_2_O_2_ after plasma treatment (Figure 3). To validate the specificity of the H_2_O_2_ assay, the detection of H_2_O_2_ was first evaluated across a concentration range (0–1000 μM) in the presence and absence of catalase (Figure 3a). While mock solutions of H_2_O_2_ in SIII supplemented with tyrosine (1.7 mM), not plasma-treated, showed a linear recovery of H_2_O_2_, the addition of catalase (15 U/ml) completely suppressed the signal, confirming assay specificity (Figure 3a). Furthermore, the addition of increasing catalase concentrations (0–180 U/mL) to PTWS prepared from SIII supplemented with tyrosine resulted in a modest, concentration-dependent reduction of detectable H_2_O_2_ for both PTWS air plasma-treated for 3 min (air 3 min PTWS) and O_2_ for 45 s (O_2_ 45 s PTWS) as reported in Figure 3b. One-way ANOVA indicated no statistically significant difference across the tested enzymatic concentrations (ns = *p* > 0.001).

The persistent H_2_O_2_ levels observed upon catalase addition in both air- and O_2_-plasma-treated solutions suggest that H_2_O_2_formation in these systems is continuously sustained by organic solutes in the medium. However, potential interference or oxidative inactivation of the enzyme in such a highly reactive environment cannot be entirely ruled out, warranting further dedicated investigation.

Concerning NO_2_^–^ ions, the results are quite different respect to the case of H_2_O_2_, since they were abundantly generated in all tested solutions (Figure 4). Similar NO_2_^–^ amounts were generated in dd-water and in its solutions with l-cysteine (Figure 4a), nicotinamide (Figure 4c) and d-glucose (Figure 4d). The increase of concentration of these molecules did not sort any effect on NO_2_^–^ generation. On the contrary, in case of l-tyrosine, an increase of its concentration resulted in the enhancement of NO_2_^–^ generation (Figure 4b), while the increase of resorcinol concentration progressively decreased the amount of NO_2_^–^ (Figure 4e).

**Figure 4:**
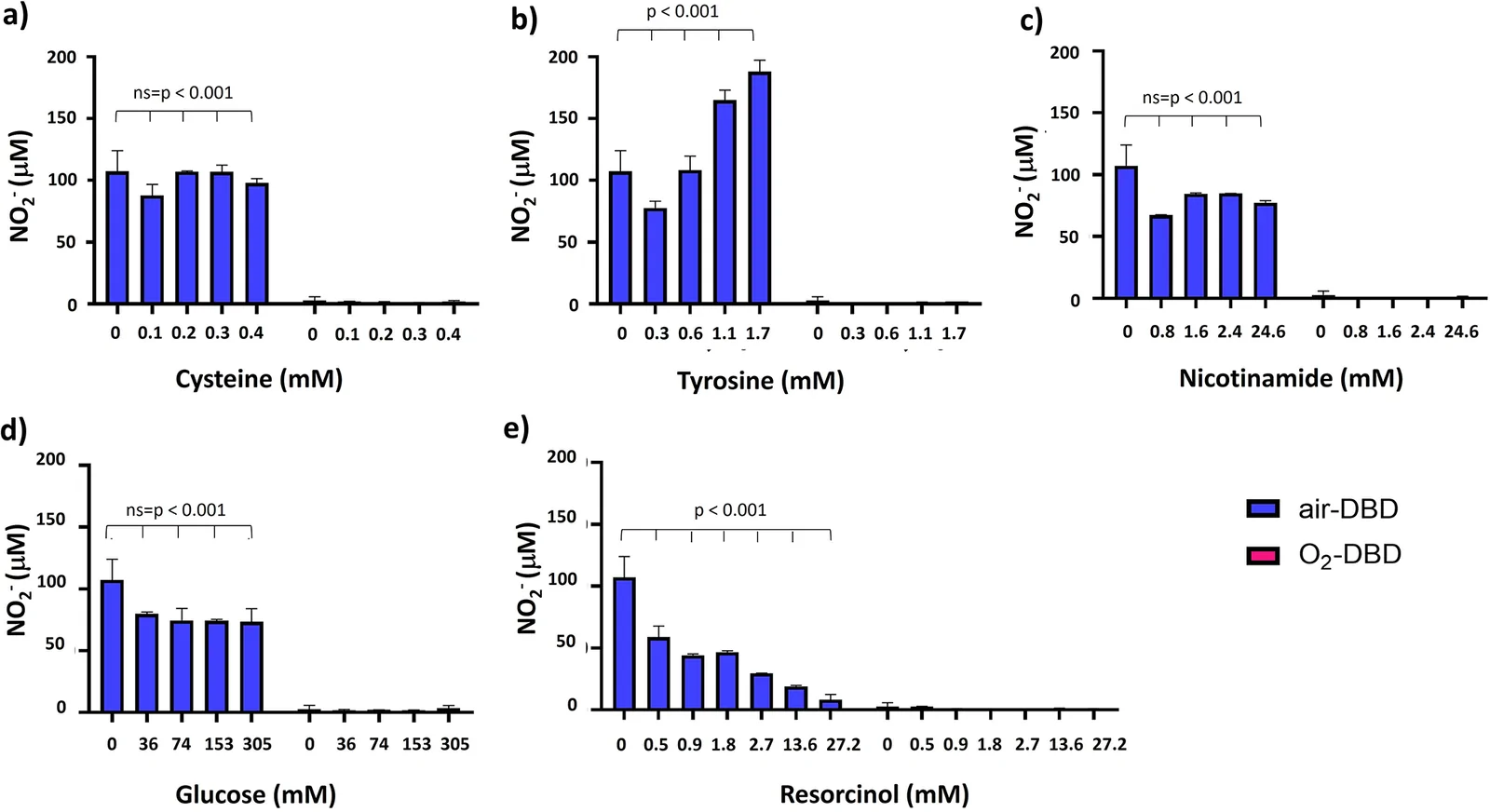
NO_2_^–^ concentration in PTWS generated in 2 mL of water solutions with increasing concentrations of a) l-cysteine (0–0.4 mM); (b) l-tyrosine (0–1.7 mM); (c) nicotinamide (0–24 mM); (d) d-glucose (0–305 mM); (e) resorcinol (0–2.72 mM) with 3 min CAP treatment (13.5 kV, 6 kHz, 25% DC, 100 ms period, 0.5 L·min^-1^) in air or O_2_ feed. The data points at 0 in the *x* axis correspond to treatments in dd-water.

Our first hypothesis is that in our conditions the formation of NO_2_^–^ ions is prevalently dependent on the chemical composition of the plasma. In fact, their generation was found abundant in all the solutions when the DBDs were fed with air, while it resulted completely inhibited when the DBDs were fed with pure O_2_, clearly lacking nitrogen precursors. These results well match the accepted mechanism for NO_2_^–^ generation in PTWS, based on the dissolution and subsequent dissociation of nitrous acid (HNO_2_), after its generation in the plasma through chemical reactions involving nitric oxide (NO), formed in plasma through the well-known Zeldovich mechanism, specifically involving O_2_ and excited N_2_.^14,18,38^ Clearly, by feeding the plasma with pure O_2_, this mechanism cannot be initiated and NO_2_^–^ is not produced in the treated liquids, at all concentrations of added organic molecules.

In the case of l-tyrosine, though, it is possible that reactions of plasma-diffused RONS involving the organic molecule could additionally contribute to NO_2_^–^ generation through reaction pathways still unknown. The apparent ‘excess’ accumulation of nitrites in the presence of tyrosine is presumably a consequence of radical-scavenging kinetics. In pure water, before and after plasma treatment and in water solutions of organic molecules treated in air plasma, plasma-generated nitrites are continuously consumed by primary aqueous oxidants like hydroxyl radical and peroxynitrite ions due to the acid pH (Figure S8).^39^ Tyrosine we suppose rapidly scavenges these primary radicals (k_OH+Tyr_ ∼ 1.3 × 10^10^ M^–1^·s^–1^),^40^ effectively protecting nitrites from being oxidized to nitrates. This competitive preservation leads to a higher net steady-state concentration of nitrites. In case of resorcinol, instead, it is rather possible that the molecule acts as a scavenger for NO_2_^–^ in solution, or for its chemical precursors (i.e., NO), since NO_2_^–^ abatement resulted clearly correlated to the concentration of resorcinol.

The data obtained from nitrites and H_2_O_2_ quantification are consistent with our previous work^27^ that reports preliminary results on tyrosine and cysteine solutions as well as on a solution of different amino acids (glycine, l-alanine, l-asparagine, l-aspartic acid, l-glutamic acid, l-proline, l-serine). Similarly, to what has been observed in the present research, in the previous paper we have found an active role of tyrosine in producing both NO_2_^–^ and H_2_O_2_, in different media including infusion solutions,^33^ differently from cysteine. Moreover, in another previous study, we also demonstrated that cysteine is involved in the scavenging of NO, the main precursor of nitrites in PTWS, probably through the formation of a labile nitro-thiol bond.^12^ In case of plasma-treated glucose solutions, instead, we only observed that the production of NO was less abundant than in dd water, thus, assuming a slight scavenging of NO by treated glucose.^12^ Measurements of pH conducted both with litmus paper and pH-meter showed that the bulk solution pH remained near neutral (pH ∼ 6–7) throughout O_2_ plasma exposure regardless of the liquid matrix (dd water or organic aqueous solutions) or operating conditions (O_2_ feed gases, treatment times up to 3 min); when Air is used as gas feed the pH falls down to 4 with PTW showing the highest acid character (pH = 2). Glass-electrode pH measurements were performed within the first 3 s of immersion of the pH meter in the liquid to avoid a progressive, artificial pH drop observed in both treated and untreated organic solutions (with the sole exception of untreated pure water). This artifact was attributed to severe electrode drift and junction fouling caused by organic target molecules and reactive plasma-generated species, which altered the electrode potential and led to an erroneous readout of continuous bulk acidification.^41^

The pH measurements reported in Figure S8 and carried out within the first 3 s of immersion lead to results consistent with litmus paper indicator. Tests performed with litmus paper confirmed that the pH remains near neutrality (pH ∼ 6–7) across all tested solutions (i.e. dd-water and water solutions) when treated in O_2_ plasma, whatever the duration of plasma processing in the range investigated (Figure S8). When plasma treatment is carried out with Air as the feed, the pH becomes acid due to the unhindered formation of acid species (i.e. HNO_2_, HNO_3_). Due to the intrinsic buffering capacity provided by the organic anions present in SIII (such as acetate and citrate), the pH of the SIII solution remains stable around its initial physiological value even after CAP exposure, as previously reported in ref 33. Importantly, maintaining a near-neutral pH after O_2_ plasma processing, rules out metal-catalyzed redox pathways (e.g., Fenton-like chemistry) as primary mechanisms for RONS formation in our system.^42^ Although Fenton-like pathways cannot be entirely ruled out in acid media (e.g., unbuffered Air-plasma-treated solutions), metal-catalyzed redox processes are expected to be minimal due to the remote plasma configuration avoiding electrode sputtering. Furthermore, the strong linear dependence of H_2_O_2_ production on the concentration of tyrosine and resorcinol (Figure 2b and e) indicates that organic-mediated pathways, rather than background trace metal catalysis, primarily govern RONS dynamics.

It is worth noting that the accumulation kinetics of plasma-generated species in the liquid phase do not always scale linearly with treatment conditions, often reaching saturation or plateau levels instead.^43^ This behavior is strongly modulated by the initial chemical composition of the liquid matrix (i.e., dissolved gases, organic solutes, and buffer components), which can act as scavenger or limit precursor availability, thereby establishing a dynamic equilibrium between formation and secondary decomposition reactions, as previously demonstrated. Furthermore, as shown in Figure 5, shifting the feed gas composition above an O_2_/(O_2_+N_2_) ratio of 0.6 yields only minor changes in final H_2_O_2_ yields for plasma-treated cysteine or glucose solutions. In contrast, significant differences were observed for plasma-treated tyrosine solutions, which reached a plateau only at an O_2_/(O_2_+N_2_) ratio of 0.9. This confirms that beyond a minimal threshold of gaseous precursors, liquid-phase chemistry and solute interactions dictate the saturation plateau.

**Figure 5:**
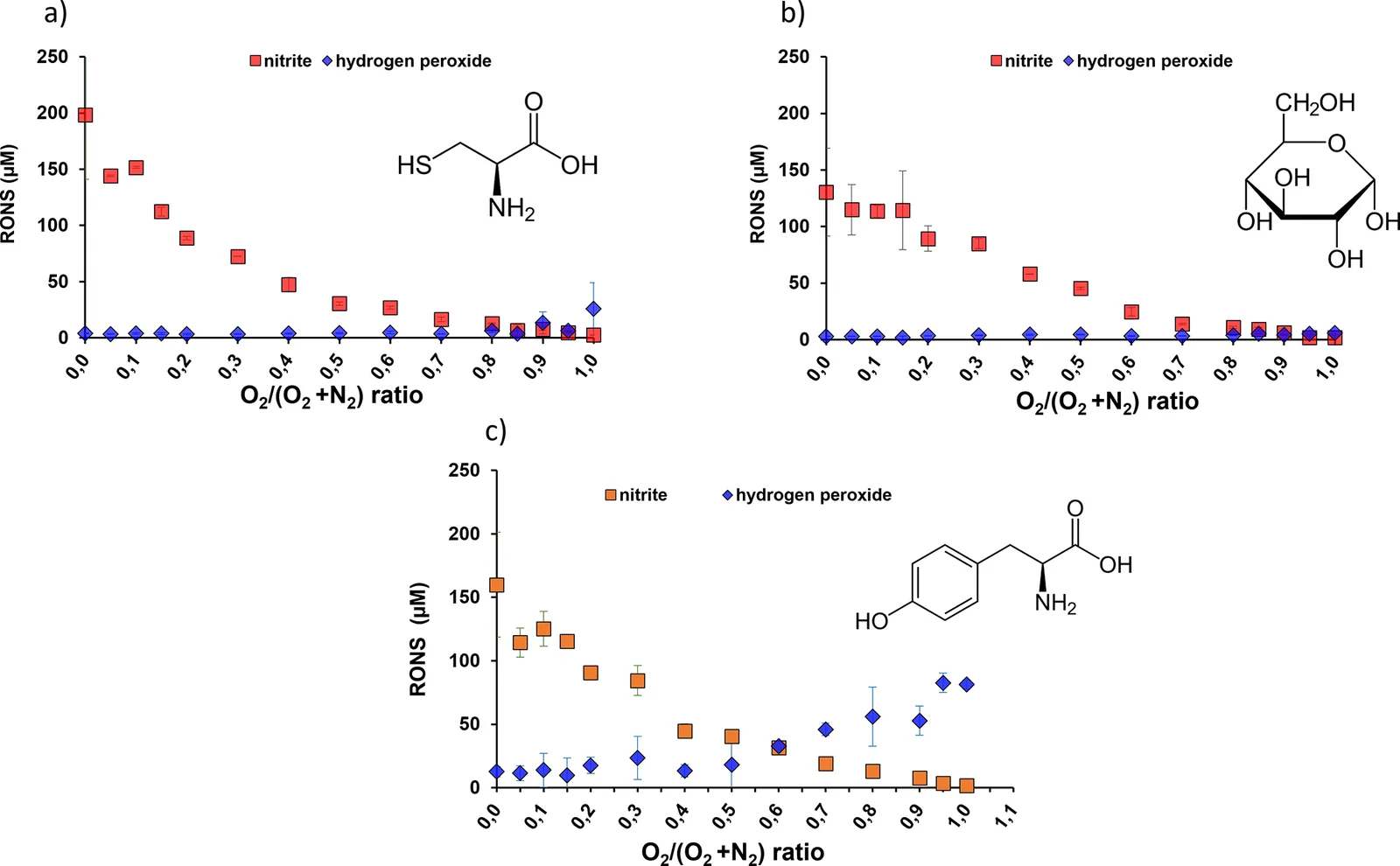
Hydrogen peroxide and nitrite concentrations in a) plasma-treated cysteine, b) plasma-treated glucose, and c) plasma-treated tyrosine as a function of O_2_/O_2_ + N_2_ feed ratio. All DBDs were ignited in the air (0.5 slm) at 13 kV, 3 mm treatment distance, far from 2 mL of liquid, for 3 min.

### 3.2 High-Resolution Mass Spectrometry Analyses

In order to better investigate the chemical modifications of organic molecules in PTWS and to eventually assess the involvement of such molecules in the formation of additional RONS, we prepared water solutions of l-cysteine (0.41 mM), l-tyrosine (1.65 mM), d-glucose (36 mM), and resorcinol (27 mM) to be analyzed by a high-resolution mass spectrometer before and after plasma treatments fed with O_2_ or air. Nicotinamide was not analyzed before and after the plasma treatment because the content of plasma-generated RONS seems to be unaffected by the presence of such biomolecule within the concentration range investigated (Figures 2 and 4). Finally, to extend the study to clinically approved and buffered solutions, SIII solutions, before and after supplementation with l-tyrosine (3.3 mM), were analyzed and compared with samples treated using O_2_- or air-fed plasmas. The tyrosine concentration in the SIII solution was deliberately increased to values exceeding 1.7 mM. This choice was motivated by previous findings, where no unambiguous evidence of tyrosine modification was detected, most likely due to instrumental interferences and signal overlap originating from other constituents of the solution, as well as their adducts and reaction products.^33^ Increasing the tyrosine concentration is therefore expected to improve signal discrimination, reduce the relative impact of interfering species, and enable a more reliable identification of structural modifications affecting the target molecule. Mass fragmentation pattern for tyrosine subproducts and theoretical exact masses for the analyzed compounds are compared with the experimental values, accurate to four decimal digits, in Section 2.3 of the Supporting Information.

#### 3.2.1 Plasma Modification of L-Cysteine Solutions

Figure S1 shows the positive mass spectra of l-cysteine solution acquired before and after plasma treatment in air and O_2_. The peak of cystine (two disulfide-linked cysteines) at 241.0294 *m*/*z* is well visible in untreated as well as in both treated solutions (Figure S1). The formation of disulfide bonds is well established as a spontaneous process mediated by molecular oxygen through production of cysteine sulfenic acid.^44^ For instance, under aerobic conditions, a thin layer of cystine is expected to be generated at the air–liquid interface when a cysteine solution is left exposed to air. Adduct of cystine with sodium and potassium ions were respectively found at 263.0109 *m*/*z* and 278.9780 *m*/*z* in the positive ion mode treated solutions (Figure S1b and c). From the spectra, it seems that plasma treatments, in the experimental conditions used, do not alter the chemical structure of cysteine. Similar results were obtained by increasing the treatment times with both feeds up to 5 min (data not shown).

These results confirm that cysteine does not contribute neither to the synthesis of H_2_O_2_ nor to the formation of NO_2_^–^, thus justifying the absence of a correlation between the concentration of such RONS and that of cysteine in solutions, as reported in Figures 2a and 4a.

A possible formation route of cystine in PTWS is reported in Scheme S1.^45^

#### 3.2.2 Plasma Modifications of D-Glucose Solutions

The negative mass spectra acquired for untreated and treated glucose solutions are reported in Figure S2. The untreated glucose molecule is found at 215.0304 *m*/*z* as adduct with a chloride ion [Glucose+Cl]^−^, in negative ion mode. The molecular structure of d-glucose appears retained in the case of solutions plasma treated in both air and O_2_ feeds at lower treatment times (up to 5 min), as shown in Figure S2b and c. By increasing the treatment time of air-DBD up to 15 min, instead, the molecular structure of d-glucose appear nitrated (Figure S2d): the peak of [Glucose+Cl]^−^ adduct is not visible in the spectrum, while other three main peaks at 240.8967 *m*/*z*, 225.9222 *m*/*z* and 209.9566 *m*/*z* are clearly visible, attributed to hydrated dinitro-glucose, nitro-gluconic acid, and nitro-glucose, respectively. The presence of nitro derivatives at high treatment times justifies the tendency of plasma-treated glucose to act as a scavenger of NO precursors in solution, according to our previous findings, where the signal of NO in PTWS was significantly reduced in the presence of d-glucose.^12^ In any case, the retained integrity of the chemical structure at low treatment times (up to 3 min) confirms that the molecule is not involved in interactions with plasma-produced RONS in these conditions. This result agrees with the trend observed for H_2_O_2_ and NO_2_^–^ in the solutions, found almost independent of the concentration of d-glucose in the liquid (Figures 2d and 4d). Our results confirm that long plasma treatments in O_2_ promote the oxidation of glucose;^46^ in addition, however, we demonstrate that when air is used as feed nitro-derivatives are formed.

#### 3.2.3 Plasma Modifications of L-Tyrosine Solutions

In Figures S3 and S4, the MS spectra acquired, respectively, on l-tyrosine solutions untreated and treated with O_2_- and air-DBDs at different treatment times are reported. In Figures S5, S6 and S7, respectively, mass fragmentation patterns are reported for species “5” and “8” shown in Figure 6, and species 4 in Figure 7.

**Figure 6:**
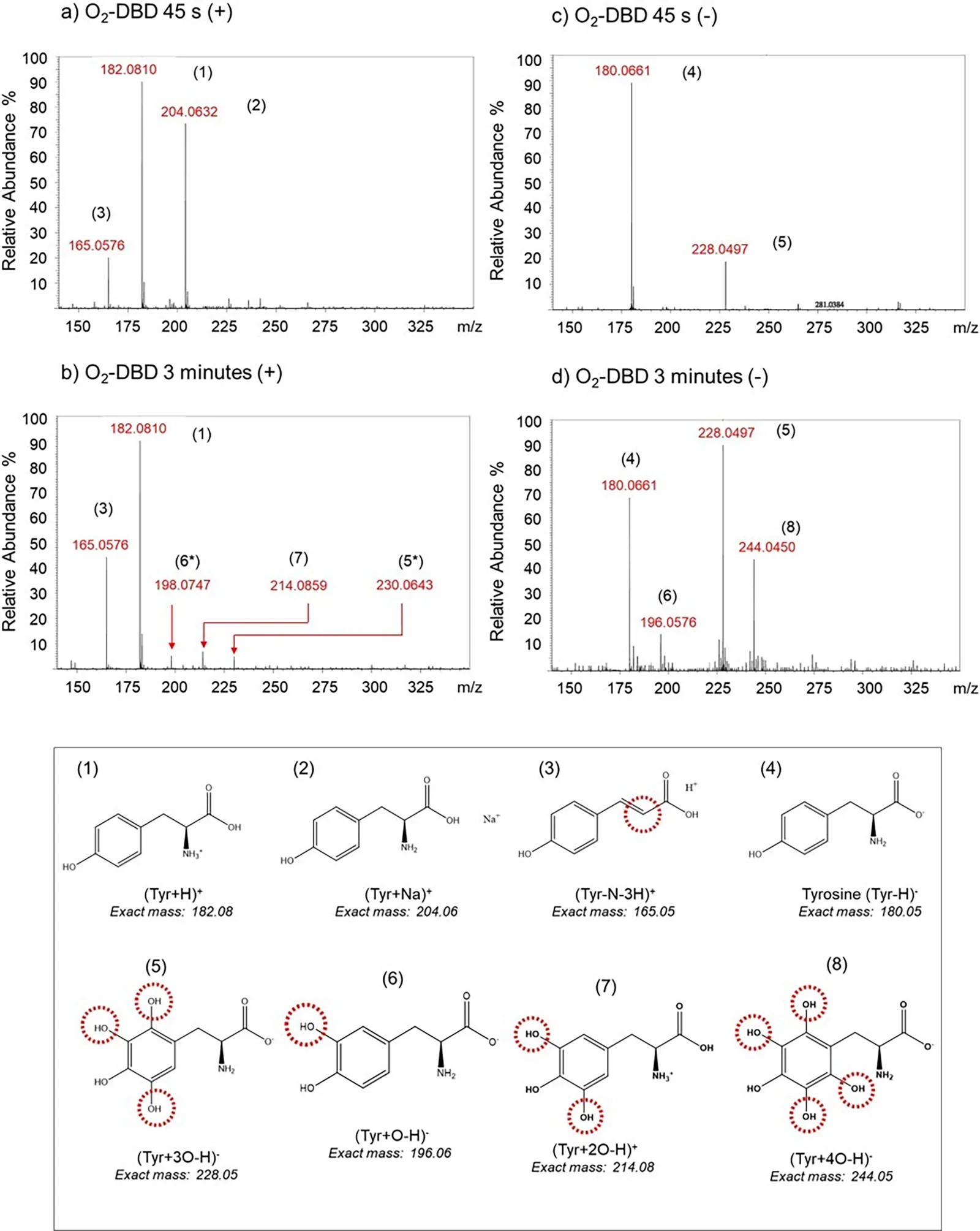
MS spectra in positive (a–b) and negative (c–d) ion mode of l-tyrosine solution (1.7 mM) after (a–c) 45 s and (b–d) 3 min O_2_-DBDs ignited at 13.5 kV, 6 kHz, 25% DC, 100 ms period, 0.5 L·min^-1^ flow rate. The proposed molecular structures for the most abundant peaks (1)–(8) are shown. 5* and 6* are the protonated species deriving from 5 and 6.

**Figure 7:**
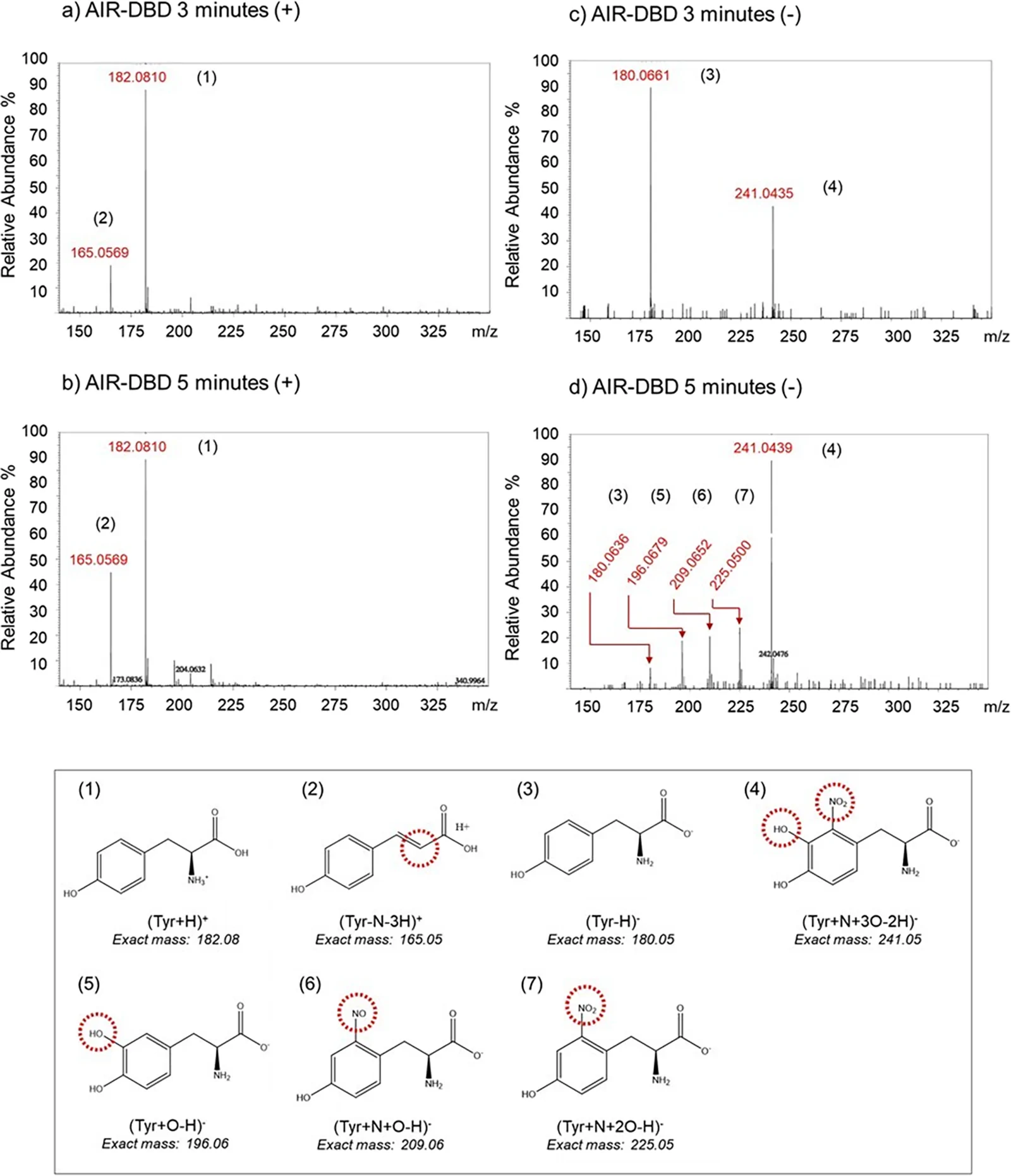
Positive (a–b) and negative (c–d) mass spectra of l-tyrosine (1.7 mM) after treatment in 3 min (a–c) and 5 min (b–d) air-DBDs ignited at 13.5 kV, 6 kHz, 25% DC, 100 ms period, 0.5 L·min^-1^ flow rate. The molecular structures proposed for the most abundant peaks (1)–(7) are shown.

In Figure S3, the characteristic peaks of the untreated tyrosine solution are observed in positive-ion mode at *m*/*z* 182.0810 and 204.0622 (Figure S3a), corresponding to protonated tyrosine ([Tyr+H]^+^) and its sodium adduct ([Tyr+Na]^+^), respectively. In negative-ion mode, the molecule is detected in its deprotonated form ([Tyr–H]^−^) at *m*/*z* 180.0661 (Figure S3b).

Concerning the modification of l-tyrosine in O_2_-DBDs after 45 s and 3 min of treatment, the MS spectra in Figure 6Figure [Fig fig4] report that for both times many molecular hydroxylation products are formed, with progressively increasing number of hydroxyl groups inserted in the aromatic ring: [Tyr+O+H]^+^ at 198.0747 *m*/*z* in the positive (Figure 4b) and [Tyr+O–H]^−^ at 196.0576 *m*/*z* in the negative mode (Figure 6d); [Tyr+2O+H]^+^ at 214.0859 *m*/*z* in the positive mode (Figure 6b); [Tyr+3O+H]^+^ at 230.0643 *m*/*z* in the positive (Figure 6b) and [Tyr+3O–H]^−^ at 228.0497 *m*/*z* in the negative mode (Figure 6c and d); [Tyr+4O–H] ^–^ at 244.0450 *m*/*z* in the negative mode (Figure 6d). In addition, we have also found a fragmentation product at 165.0576 *m*/*z* in the positive ion mode (Figure 6a and 6b), with loss of the −NH_2_ group, corresponding to [Tyr-N-3H]^+^.

As previously mentioned, the MS/MS spectra of the major species generated upon plasma treatment of tyrosine under both air and oxygen plasmas have been included in the Supporting Information, as tyrosine derivatives were the only analytes present at a sufficient concentration to afford informative tandem mass spectra. Overall, these data further corroborate the structural assignments previously inferred from the exact mass measurements and isotopic pattern analyses. A concise discussion of the most significant fragmentation pathways is provided as follows.

The MS/MS spectrum of the species at *m*/*z* 228, shown in Figure S5, provides additional structural information supporting the proposed molecular assignment. The major fragment ion at *m*/*z* 184.0600 is consistent with a neutral loss of CO_2_ (44 Da), attributable to a decarboxylation process. A subsequent intramolecular cyclization involving the aminoethyl side chain is plausibly favored, leading to the formation of a stable five-membered ring system that readily undergoes dehydration (−18 Da) to yield the fragment ion at *m*/*z* 166.0487. The observed fragmentation pathway is therefore fully consistent with the proposed structure and further corroborates the structural assignment inferred from the exact mass and isotopic pattern analyses. The fragmentation of the species at *m*/*z* 244 shown in Figure S6 proceeds through two parallel pathways. One route involves dehydration to form the cyclic intermediate at *m*/*z* 226, followed by decarboxylation yielding the fragment at *m*/*z* 182. The alternative route starts with decarboxylation of the precursor ion at *m*/*z* 200, followed by loss of ethylamine to generate the fragment at *m*/*z* 156, which subsequently undergoes dehydration to produce the ion at *m*/*z* 138. The occurrence of these complementary fragmentation channels strongly supports the proposed molecular structure.

The spectrum of the species at *m*/*z* 241, shown in Figure S7, is characterized by two major fragment ions at *m*/*z* 194.0450 and 150.0536. The fragmentation pathway is initiated by the loss of a nitrate anion, followed by an intramolecular cyclization accompanied by dehydration (−48 Da), leading to the formation of the stable cyclic intermediate at *m*/*z* 194.0459. Subsequent decarboxylation (−44 Da) provides the fragment ion at *m*/*z* 150.0561. The observed fragmentation pattern is fully consistent with the proposed structure and provides additional support for the molecular assignment inferred from the HRMS analyses.

Our results are consistent with previous reports showing that the aromatic ring of tyrosine is readily hydroxylated upon plasma exposure,^30^ although differences emerge compared with other tyrosine formulations.^33^ The products observed in Figure 6 are likely initiated by plasma-generated reactive species, such as ozone and hydroxyl radicals, with hydroxylation occurring predominantly on the aromatic ring.^14^ Among the main products, dihydroxy-phenylalanine (DOPA) is detected at *m*/*z* 198.0747 (Figure 6b) and 196.0576 (Figure 6d), while hydroxyl-DOPA appears at *m*/*z* 214.0859 (Figure 6b). DOPA is a key intermediate in tyrosine metabolism and, together with tyrosine, modulates the melanocyte activity. Its clinical use in neurodegenerative disorders^47^ suggests that plasma-treated, tyrosine-rich solutions may influence not only RONS accumulation but also the DOPA/tyrosine balance, potentially enhancing treatment efficacy, an aspect relevant also for direct plasma exposure of tissues.

Additionally, in PTWS, tyrosine promotes further H_2_O_2_ formation, amplifying the overall effect. This behavior can be attributed to the intrinsic reactivity of phenolic groups, which are prone to oxidation and hydroxylation due to favorable electronic and steric factors that promote its reaction with peroxyl or alkoxyl radicals.^48^ Based upon this literature, the proposed mechanism of the autoxidation of a phenol like tyrosine and resorcinol is illustrated in Scheme 1. The generation of a quinone in the presence of peroxynitrite and/or NO_*x*_ species like NO_2_ or N_2_O_3_ has been hypothesized^49^ as responsible of the generation of the peroxide anion. Thus, the generated O_2_^–^ spontaneously converts to H_2_O_2_ through dismutation. Moreover, polyphenols can be also oxidized by the generated O_2_^–^ to yield the semiquinone radical and H_2_O_2_. This process can be initiated also by atmospheric O_2_, although the rate of this reaction would be low due to the low O_2_/O_2_^–^ redox potential and spin restriction between the reactants.^50^ In many papers it is reported, in fact, that the addition of phenols and polyphenols in cell culture media led to spontaneous generation of H_2_O_2_ over time.^51,52^ In case of plasmas containing ROS much stronger than O_2_, this process is clearly amplified. This assumption is also supported by the evidence that the overproduction of H_2_O_2_ in treated tyrosine solutions was higher in O_2_ than in air (Figure 2b). In principle, such mechanism should involve all molecules with phenolic rings exposed to oxygen-based plasmas. In case of plasma fed with air another mechanism like that reported in Scheme 1 can be invoked, where the formation of the hydroquinone passes through the reaction of the phenol with peroxynitrite.^49^

**Scheme 1:**
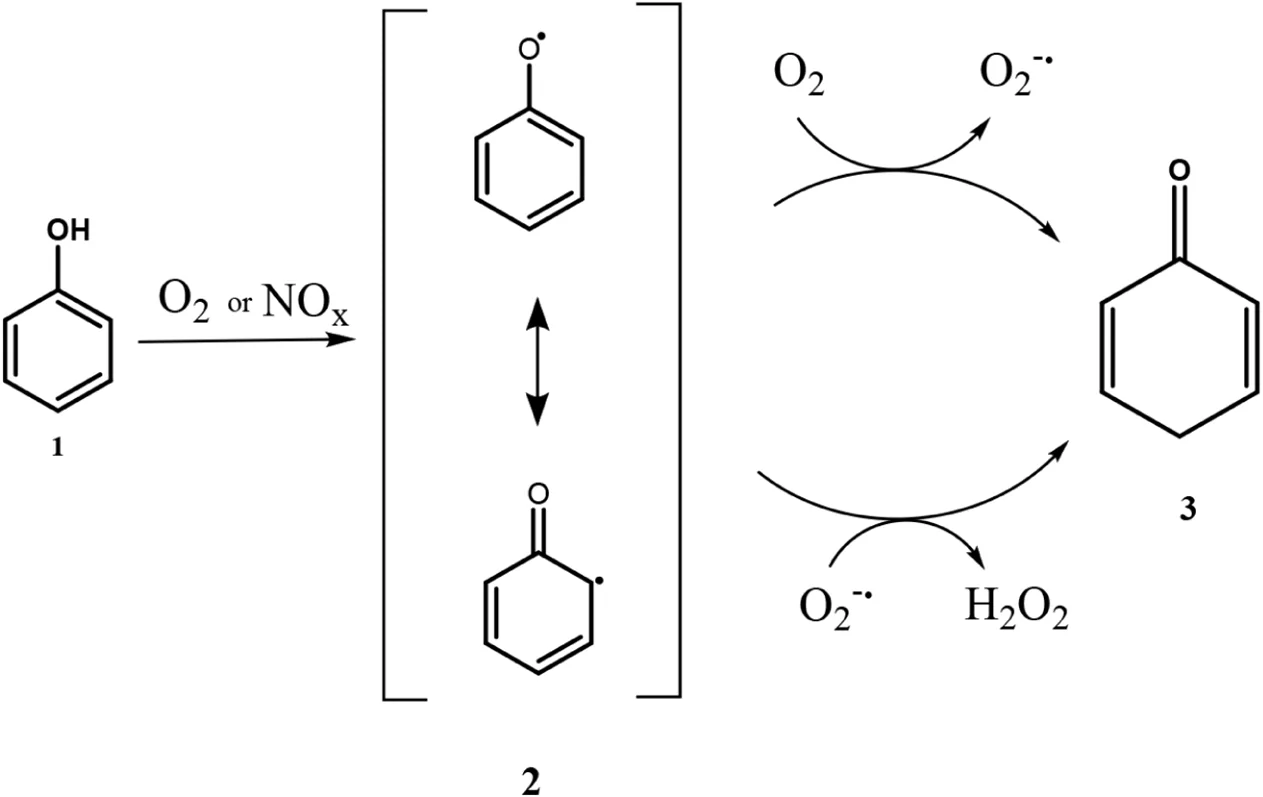
Possible Mechanism for H_2_O_2_ Generation in PTWS Containing Organic Molecules with Phenolic Functional Groups

Similarly to hydroxylation, nitration is also favored in the presence of phenolic rings.^50^ The modifications of l-tyrosine solutions after 3 and 5 min exposure to air-DBD are shown in Figure 7, where mass spectra reveal several derivatives, including physiologically relevant nitro-tyrosine. Specifically, three nitro-derivatives were detected: [Tyr+N+O]^−^ at 209.0652 *m*/*z* (Figure 7d), [Tyr+N+2O]^−^ at 196.0679 *m*/*z* (Figure 7d), and [Tyr+3O+N+H]^+^ at 241.0439 *m*/*z* (Figure 7c and 7d). Their formation increases with treatment time (Figure 7c vs [Fig fig7]d). As these species are absent in O_2_-DBD conditions (Figure 6), their origin can be attributed to reactions with plasma-generated RNS, which are more abundantly produced in air-fed discharges.

As reported by L. Grossi,^49^ tyrosine nitrosylation can proceed via an electron-transfer mechanism, likely mediated by NO_*x*_ species and involving the formation of a phenoxyl-type radical intermediate. Under acid conditions (pH < 4), such as those reached in PTWS derived from unbuffered dd-H_2_O supplemented with l-tyrosine exposed to air DBDs, the formation of peroxynitrite from HNO_2_ and H_2_O_2_ is favored. The presence of water in the plasma further promotes peroxynitrite generation, which can react with the aromatic ring of tyrosine to yield the nitro derivatives detected by MS. A plausible reaction pathway, along with the involved plasma-generated RNS, is outlined in Scheme 2.

**Scheme 2:**
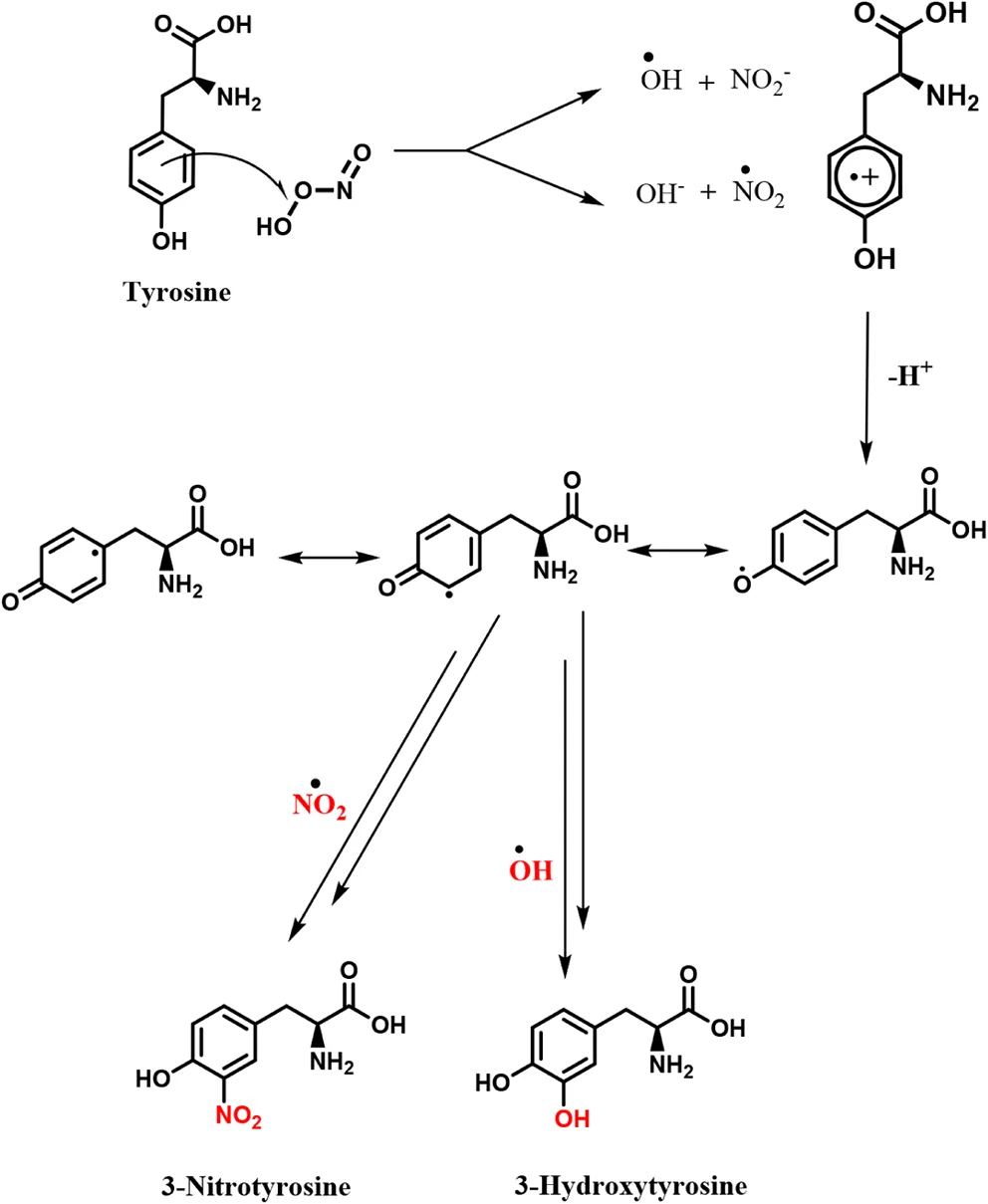
Possible Mechanism for the Generation of Nitro-Derivatives of tyrosine in PTWS from in Air-DBDs

The consumption of plasma-generated RNS in these reactions would be expected to reduce NO_2_^–^ levels in the resulting PTWS, as their formation is closely linked to the availability of NO and its derivatives.^12,27,38^ This trend is observed for resorcinol, where increasing concentration leads to a decrease in NO_2_^–^ indicating a scavenging effect (Figure 4e). In contrast, tyrosine exhibits the opposite behavior, with NO_2_^–^ concentration increasing as a function of tyrosine content, suggesting the occurrence of additional NO_2_^–^ releasing pathways. As outlined in Scheme 3, a plausible mechanism involves the amino group of tyrosine. While l-tyrosine deamination is typically enzyme-catalyzed, in PTWS it may be driven by oxidative species. In this context, a base-catalyzed elimination of the nitro group, well documented in the literature,^53^ is proposed.

**Scheme 3:**
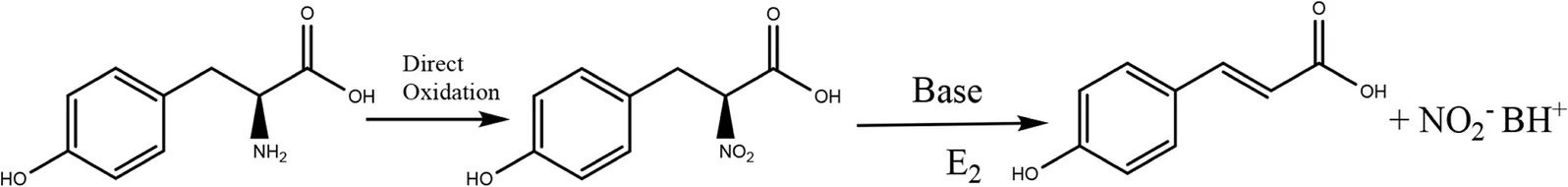
Possible Mechanism for NO_2_^–^ Generation in PTWS Containing l-Tyrosine

Upon extension of the plasma treatment time to 20 min, the formation of 3-(4-hydroxyphenyl)-2-nitropropanoic acid is observed, supporting the hypothesis that the tyrosine amino group undergoes oxidation under highly oxidative conditions (Figure S4).

The exact mass and the fragmentation pattern (Figure S4c) make the interpretation of the nature of the signal quite certain. The presence of 3-(4-hydroxyphenyl)-2-nitropropanoic acid is further evidence of the proposed mechanism that involves the formation of coumaric acid, detected at *m*/*z* 165.05 in MS spectra of dd-H_2_O tyrosine solutions both after O_2_^–^ and air-DBD treatments (Figures 6a–b and 7a–b). The formation of coumaric acid could derive from the extraction of the amino group and the consequent formation of a double bond. This reaction, followed by oxidation of the amino group,^54^ could produce nitrite ions as well. Oxidation preferentially targets the aromatic ring; however, in the strongly oxidative environment of O_2_-DBD, NO_2_^–^ it is likely further oxidized to NO_3_^–^, consistent with the absence of detectable nitrite under these conditions (Figure 4b). Conversely, in air-fed discharges, competition between ROS and RNS may limit complete oxidation, allowing NO_2_^–^ to persist and enabling additional formation pathways beyond HNO_2_ dissolution from the plasma. Notably, this mechanism is not operative for resorcinol, which lacks an amino group and therefore primarily acts as a nitrite scavenger.

In any case, besides the effect on RONS generation, most of the nitro derivatives of tyrosine induced by plasma-generated RNS could concur to determine the biological impact of the resulting PTWS.

Beyond their impact on RONS chemistry, plasma-induced tyrosine derivatives, including nitro compounds, are likely to contribute to the biological effects of PTWS. Coumaric acid is a natural metabolite contained in many edible plants, and its antioxidant activity in reducing oxidative stress and inflammatory reactions is well recognized.^55^ These considerations indicate that the presence of such reaction products should not be overlooked in biological assays, as they may significantly influence observed outcomes. Overall, the wide range of plasma-induced transformations of biomolecules in PTWS highlights the complexity of these systems.

#### 3.2.4 Plasma Modifications of Resorcinol Solutions

The mass spectra acquired on resorcinol solutions (27.2 mM) after exposure to air- and O_2_-DBDs are shown in Figures 8 and 9, respectively. The peak of untreated resorcinol is found at 111.0432 *m*/*z*, in the protonated form as [Res+H]^+^, in positive-ion mode (Figure 8a). Concerning the treatment in O_2_-DBD, similar to the case of tyrosine, mass spectra report the formation of hydroxyl derivatives, mostly through aromatic ring hydroxylation, corresponding to [Res+O+H]^+^ at 127.0569 *m*/*z* (Figure 8b) and [Res+2O+H]^+^ at 142.0289 *m*/*z* (Figure 8b). Similarly to tyrosine, in fact, the presence of resorcinol in solution proved to increase the concentration of H_2_O_2_ generated in the resulting PTWS, proportionally to the concentration of resorcinol (Figure 2b and e). As in the case of tyrosine, we assume that the oxidation of the molecule through the quinone pathway assumed in Scheme 1 is responsible for H_2_O_2_ overproduction in the presence of the molecule. While transient radical intermediates (e.g., phenoxy and semiquinone radicals) were not directly detected, their involvement represents a mechanistic hypothesis fully consistent with the stoichiometric balance and the characteristic oxidation byproducts identified by LC-MS.

**Figure 8:**
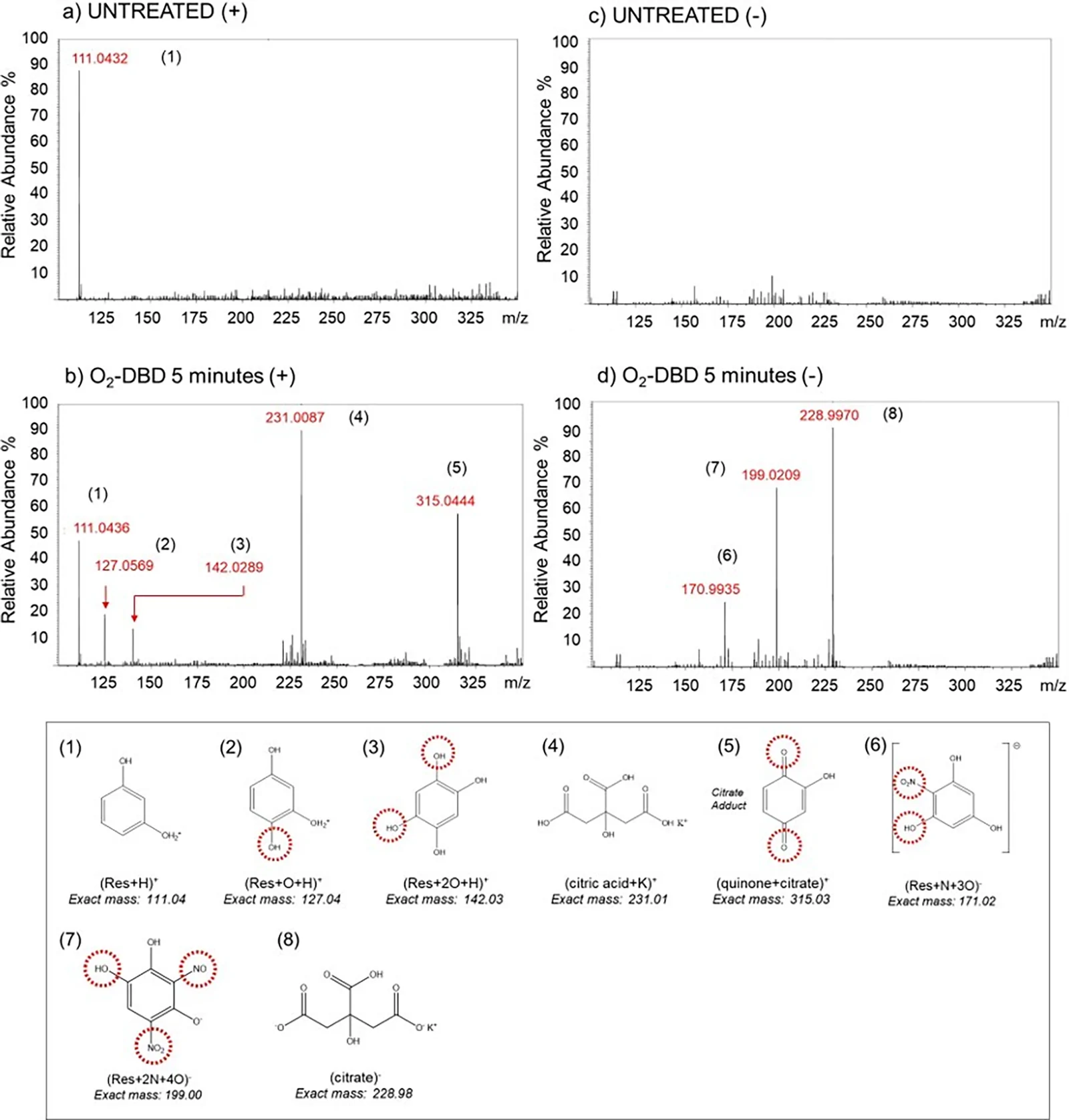
Mass spectra of resorcinol (27.2 mM) in positive (a–b) and negative (c–d) ion mode: (a–c) untreated and (b–d) after 5 min O_2_-DBDs ignited at 13.5 kV, 6 kHz, 25% DC, 100 ms period, 0.5 L/min flow rate. The molecular structures proposed for the most abundant peaks (1)–(8) are shown.

**Figure 9:**
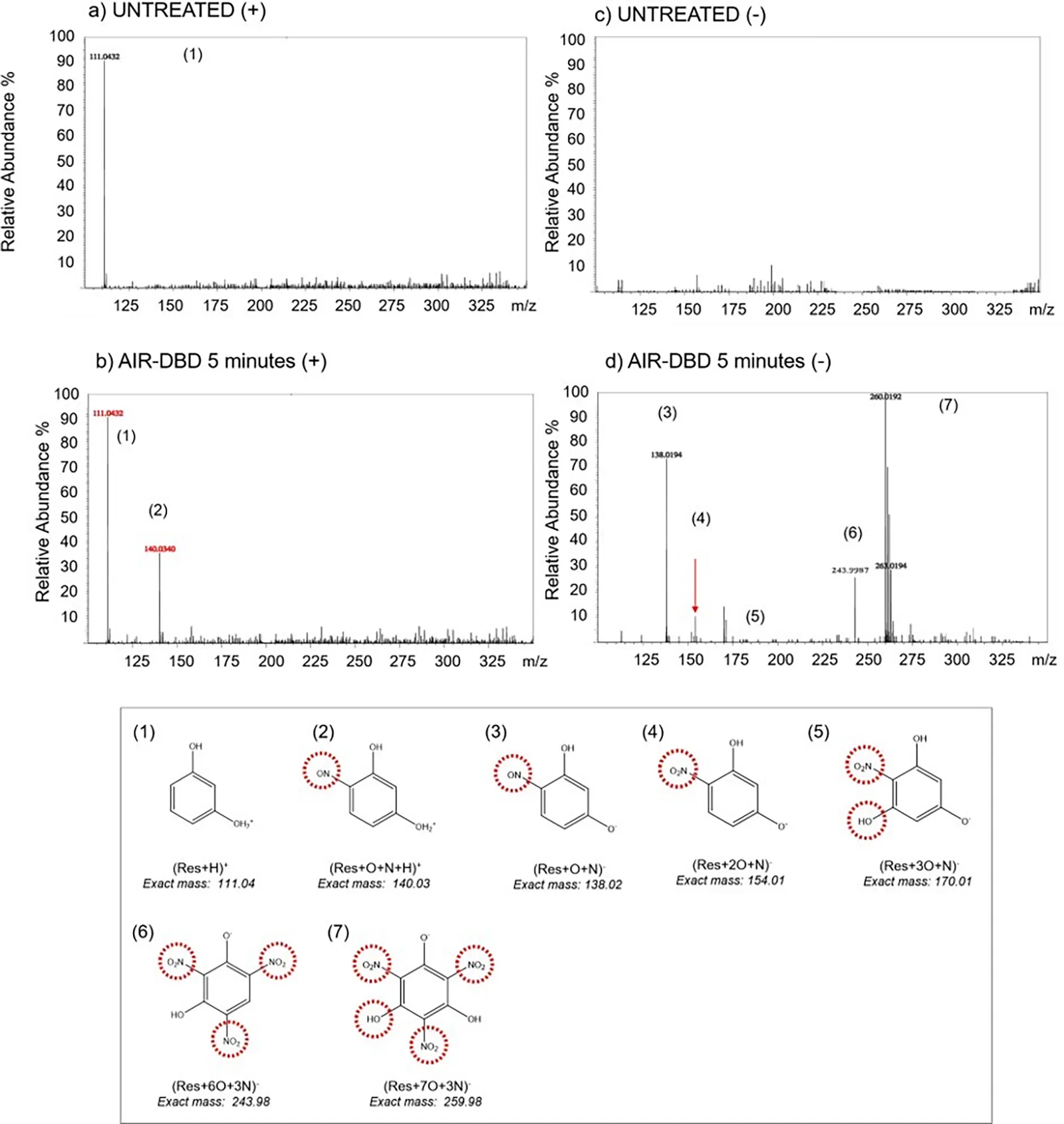
Mass spectra of resorcinol (27.2 mM) in positive (a–b) and negative (c–d) ion mode: (a–c) untreated and (b–d) after 5 min air-DBDs ignited at 13.5 kV, 6 kHz, 25% DC, 100 ms period, 0.5 L·min^-1^ flow rate. The molecular structures (1)–(8) proposed for the most abundant peaks are shown.

Further evidence in support of this mechanism is the presence of a quinone at 315.0444 *m*/*z*, as an adduct with citrate (Figure 8b). This form of quinone is also reported to generate H_2_O_2_ in vivo.^56^

Another important product of resorcinol from O_2_-DBD, in fact, is citric acid, found at 231.0087 *m*/*z* in the positive (Figure 8b) and at 228.9970 *m*/*z* in the negative ion mode (Figure 8c), possibly formed through ring oxidation and opening reactions. Interestingly, in the negative ion mode, it is possible to also identify nitro-derivatives of resorcinol, [Res+3O+N]^−^ at 170.9935 *m*/*z* and [Res+2N+4O] ^–^ at 199.0209 *m*/*z*. This last result is not expected while working in pure O_2_ feed; we can assume that residual air in the headspace of the liquid could introduce traces of N_2_ that form RNS in the plasma phase, which are rapidly scavenged by resorcinol in solution, generating the above-mentioned nitro-derivatives. This assumption would also confirm the strong ability of resorcinol to scavenge RNS from the plasma, as discussed in Section 3.1.

In fact, mass spectra acquired on resorcinol solutions exposed to air-DBD (Figure 9) confirm the ability of the molecule to undergo single and multiple nitrosylation at the aromatic ring. The most abundant products, in fact, are generated by addition of increasing numbers of nitro- and nitric- groups, corresponding to [Res+O+N+H]^+^ at 140.0340 *m*/*z* (Figure 9b), and [Res+N+O]^−^ at 138.0194 *m*/*z*, [Res+2O+N]^−^ at 145.0169 *m*/*z*, [Res+3O+N] ^–^ at 170.0097 *m*/*z*, [Res+6O+3N]^−^ at 243.9987 *m*/*z* and [Res+7O+3N]^−^ at 260.0192 *m*/*z*, (Figure 9c). It is our opinion that the formation of all these nitro-derivatives from resorcinol treated with air-DBDs could be responsible for the scavenging of the same plasma-generated RNS involved in the generation of nitrite ions (like ONOO^–^ and NO). This would explain the trend observed for nitrites detected as a function of resorcinol concentration (Figure 4e).

#### 3.2.5 Plasma Modifications of Infusion Solution (SIII) Supplemented or Not with Tyrosine

The spectra acquired in SIII solution supplemented or not with tyrosine both before and after plasma treatment in O_2_ or air-fed plasma are reported in Figure 10.

**Figure 10:**
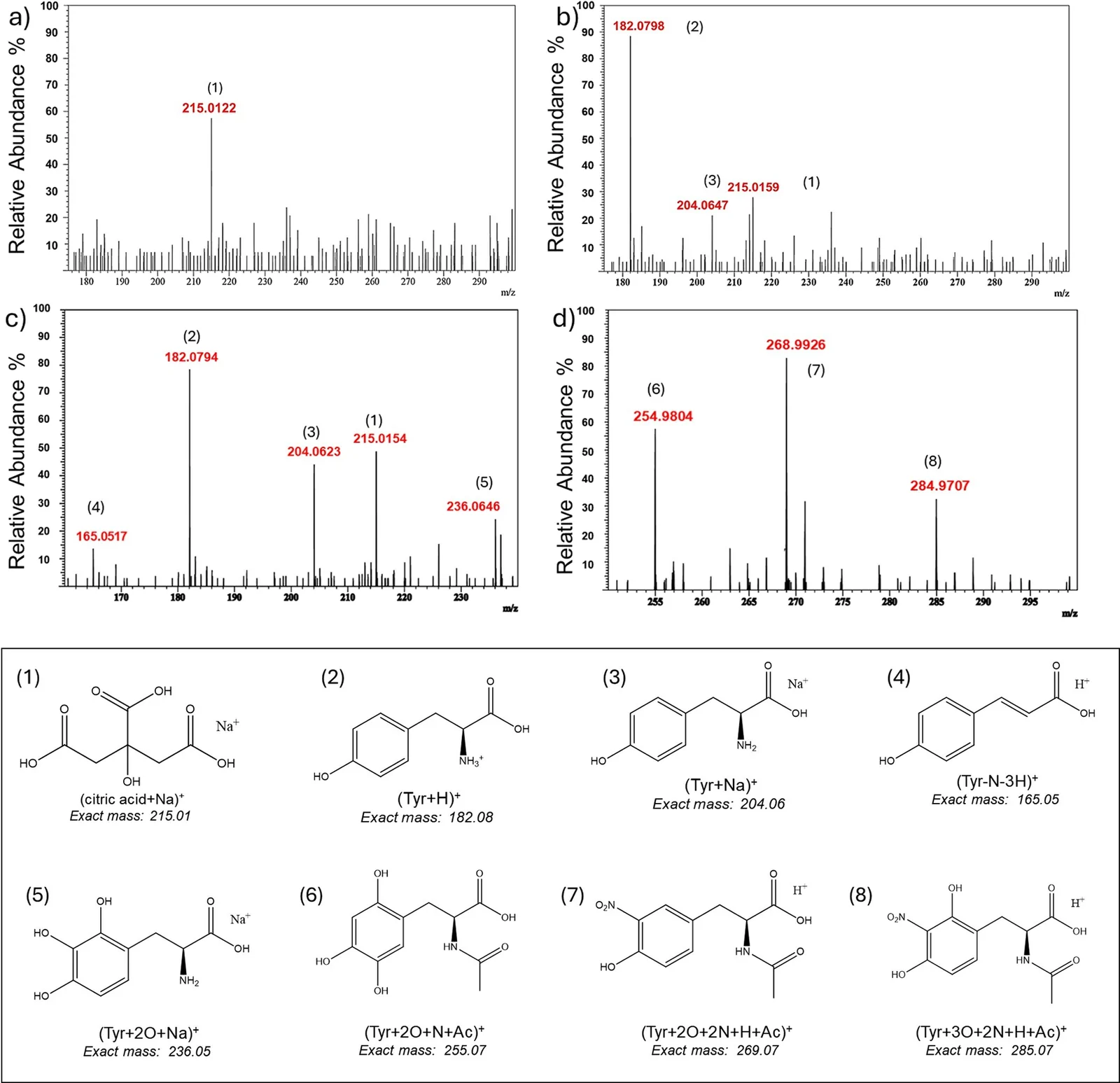
Mass spectra of a) SIII solution, b) SIII+ l-tyr (3.3 mM), c) SIII+ l-tyr (3.3 mM) treated with O_2_ plasma, and d) SIII+ l-tyr (3.3 mM) treated with air plasma in positive ion mode. DBDs were ignited on 30 mL of SIII-Tyr solution at 10 kV, 9 kHz, 25% DC, 100 ms period, 1 L·min^-1^ gas feed flow rate. The molecular structures on the right side of each spectrum are proposed for the most abundant peaks shown.

As it can be observed from the spectra reported in Figure 10, the SIII solution (Figure 10a) shows the presence of a peak at *m*/*z* 255.0122, which can be attributed to citrate. This signal is also detected in the SIII solution supplemented with tyrosine (Figure 10b), where additional peaks at *m*/*z* 182.0798 and 204.0647 are observed, corresponding to protonated tyrosine and its sodium adduct, respectively.

Following treatment with O_2_-fed plasma (Figure 10c), the formation of oxygenated tyrosine derivatives was evident. This behavior is consistent with the previous observation in simpler aqueous systems containing tyrosine and can be rationalized by oxidative pathways involving deamination, leading to products such as p-coumaric acid. These findings indicate that analogous reaction mechanisms operate, even in more complex matrices.

The increase in H_2_O_2_ production with tyrosine concentration in SIII solutions,^33^ similar to the trend observed in dd H_2_O_2_-Tyr systems, suggests that probably the same mechanisms of formation of H_2_O_2_ act in both media.

Air plasma treatment results in both nitrosylated and oxidized derivatives of the tyrosine aromatic ring. Additionally, condensation byproducts involving acetate are detected, likely arising from reactions with the amino group of tyrosine, yielding the corresponding amides at 254.9804 *m*/*z*, 268.9926 *m*/*z* and 284.9707 *m*/*z*. Finally, acetate and citrate anions present in SIII may also contribute to NO_2_^–^ formation (Scheme 3) by acting as bases.

While single-component solutions were fundamental to deconvoluting individual plasma-chemical pathways, the results obtained with the SIII mixture highlight the occurrence of plasma-assisted cross reactions between organic and inorganic species. This synergy not only validates the dominant role of molecules like tyrosine in complex environments but also underlines the broader potential of plasma-assisted organic synthesis in the liquid phase.

## 4 Conclusions

We tested the interaction of organic molecules in water solutions, l-cysteine, l-tyrosine, nicotinamide, d-glucose and resorcinol, with CAP. These molecules were chosen among a long list of amino acids, vitamins or nutrients typically present in the liquids used to generate PTWS for biomedical applications, especially for in vitro experiments. So far, the interactions of these organic components with plasma-generated RONS were underestimated, by assuming a passive diffusion of RONS into the liquid, but we show that this assumption is wrong. By exposing water solutions with increasing concentrations of those molecules to a remote treatment with a planar-DBD device, RONS analyses (H_2_O_2_ and NO_2_^–^) of the liquids revealed that organic molecules with phenolic moieties (resorcinol and l-tyrosine) could enhance the production of H_2_O_2_, while NO_2_^–^ ions resulted scavenged by resorcinol and overproduced in the presence of l-tyrosine. Mass spectrometry indicated nitrosylation and ring oxidation of organic molecules consistent with RONS overproduction or scavenging, thus allowing to hypothesize possible reaction pathways. The addition of tyrosine to more complex, clinically relevant infusion solutions (e.g., SIII) yields the same organic products, corroborating H_2_O_2_ and NO_2_^–^ formation and lending strong support to the proposed reaction mechanisms even in buffered, near-neutral pH environments. The results clearly demonstrate a synergistic interaction of organic molecules and plasma-generated RONS in the unique definition of the final composition of each resulting PTWS. Similar interactions can occur also in direct plasma treatments of cells or living tissues, as well as during the treatment of seeds and plants.

## Supplementary Material



## Notes

Mass spectrometry spectra, proposed fragmentation pathways, and pH measurements (DOCX)

## Abbreviations

CAPs: cold atmospheric plasmas
CDL: curved desolvation line
DBD: dielectric barrier discharge
DC: duty cycle
dd-H_2_O: double-distilled water
DOPA: dihydroxy-phenylalanine
ESI: electrospray ionization
e_solv_: solvated electrons
FDA: Food and Drug Administration
HV: high voltage
LCMS: Liquid chromatography–mass spectrometry
MS: mass spectrometry
NADH/NAD^+^: nicotinamide adenine dinucleotide
PBS: Phosphate-buffered saline
PTWS: plasma-treated water solutions
Res: Resorcinol
RNS: reactive nitrogen Species
RONS: reactive oxygen and nitrogen species
ROS: reactive oxygen species
SIII: Electrolyte Rehydration III Solution
Tyr: tyrosine
Vpp: peak-to-peak voltage
