# Supplementary material for: Tuning Molecular Transformations and Accumulation of Reactive Species in Plasma-Treated Liquids via Organic Biomolecules
Source: ACS Omega. 2026 Oct 27;11(44):68534–50. doi: 10.1021/acsomega.6c06500 (PMC13756706; doi:10.1021/acsomega.6c06500)
Supplement: Supplementary file 1 [file ao6c06500_si_001.docx]

Supporting information

Tuning molecular Transformations and Accumulation of Reactive Species in Plasma-Treated Liquids via Organic Biomolecules

*Valeria Veronico^a,^*^‡^*, Michele Casiello^b,^* ^‡^*, Loris Grossi^c^, Roberto Gristina^d^, Vincenza Armenise^a^, Pietro Favia^a,d^, Eloisa Sardella^d,*^*

^a^ Universita degli Studi di Bari Aldo Moro Dipartimento di Chimica, via Orabona 4, 70125, Bari, Italy

^b^ Istituto di Chimica dei Composti Organo Metallici Consiglio Nazionale delle Ricerche Sezione di Bari, c/o Universita degli Studi di Bari Aldo Moro Dipartimento di Chimica, Via Orabona 4, I-70126 Bari, Italy

^c^ Universita degli Studi di Bologna Dipartimento di Scienze per la Qualita della Vita, Corso d’Augusto, 237, I-47921 Rimini, Italy

^d^ Istituto di Nanotecnologia Consiglio Nazionale delle Ricerche Sede di Bari, c/o Universita’ degli Studi di Bari Aldo Moro Dipartimento di Chimica, via Orabona 4, 70126 Bari, Italy

^‡^ contributed equally

*contact author: [eloisa.sardella@cnr.it](mailto:eloisa.sardella@cnr.it); pietro.favia@uniba.it

**High resolution Mass Spectrometry of Cysteine, D-Glucose, and tyrosine solutions in double distilled water solutions before and after plasma treatment**

1. MATERIALS AND METHODS

1.1 PTWS generation in a planar Dielectric Barrier Discharge

To generate Plasma-Treated Water Solutions (PTWS) from organic solutes in water, we prepared model systems containing L-cysteine (100 mg L⁻¹), D-glucose (6.5 g L⁻¹), and L-tyrosine (300 mg L⁻¹). These concentrations were selected from a broader set of tested conditions to approximate those found in standard cell culture media. For L-tyrosine, the highest concentration was subjected to mass spectrometric analysis, as it yielded the greatest accumulation of hydrogen peroxide following plasma treatment (Fig. 2b in the main paper). PTWS were generated by mixing organic molecules with double distilled water (dd-H_2_O). Pure Cysteine (cat. N. C8755, Sigma Aldrich), Tyrosine (cat. N. T-3754, Sigma Aldrich), Nicotinamide (cat. N. N3376, Sigma Aldrich) or Resorcinol (cat. N. 38047, Sigma Aldrich) were used for this purpose. L-Cysteine and L-Tyrosine were dissolved in ultrasonic bath for 3h at 30°C, while D-glucose solutions were prepared by diluting a 5% Glucose water solution (Eurospital S.P.A.) to obtain the desired concentration.

2. Results:

High resolution Mass Spectrometry

2.1 MS of Cysteine/dd-H_2_O solutions

Figure S1 shows the positive mass spectra of L-Cysteine solutions. The cystine peak (241.0294 m/z) is clearly observed in both untreated and plasma-treated solutions, indicating that disulfide bond formation between cysteine molecules occurs spontaneously under aerobic conditions, likely via sulfenic acid intermediates. In treated samples, cystine adducts with sodium and potassium ions are also detected at 263.0109 m/z and 278.9780 m/z, respectively. Spectral analysis suggests that plasma treatment, even when extended up to 5 minutes, does not alter the chemical structure of cysteine. These findings demonstrate that cysteine does not contribute to the formation of either hydrogen peroxide or nitrite, consistent with the lack of correlation between cysteine concentration and RONS levels observed in the solutions.

Figure S1. Positive mass spectra of L-Cysteine (0.4 mM): (a) untreated, (b) treated with a 3 min air-DBD and (c) 3 min O_2_-DBD. DBDs were ignited at 13.5 kV, 6kHz, 25% DC, 100 ms period, 0.5 L /min flow rate. The molecular structures proposed for the most abundant peaks (1)-(3) are shown.

A possible mechanism of formation of cysteine radicals is shown in scheme S1.

Scheme S1: Possible reaction routes for the formation of Cystine

2.2 MS of D-Glucose/dd-H_2_O solutions

Negative ion mass spectra show native D-glucose at 215.0304 m/z as a chloride adduct ((Glucose+Cl)^-^, with its molecular structure preserved after plasma treatment in air and O₂ when treatment times shorter than 5 min are used. In contrast, prolonged air-DBD exposure (i.e. 15 min) promotes the formation of nitrated products at 240.8967 m/z, 225.9222 m/z, and 209.9566 m/z, assigned to hydrated dinitro-glucose, nitro-gluconic acid, and nitro-glucose. Overall, we can conclude that prolonged plasma exposure promotes oxidation in O₂ and nitration in air.

Figure S2. Negative mass spectra of D-Glucose (36 mM): (a) untreated and after treatment with (b) a 3 min air-DBD, (c) a 3 min O_2_-DBD (d) a 15 min air-DBD. DBDs were ignited at 13.5 kV, 6kHz, 25% DC, 100 ms period, 0.5 L/min flow rate. The proposed molecular structures for the most abundant peaks (1)-(4) are shown.

2.2 MS of untreated Tyr/dd-H_2_O solutions

Untreated tyrosine is detected at m/z 182.0810 ([Tyr+H]⁺) and 204.0622 ([Tyr+Na]⁺) in positive mode, and at m/z 180.0661 as the deprotonated species ([Tyr–H]⁻) in negative mode (Fig. S3).

Figure S3. MS spectra acquired from L-tyrosine untreated solution in the (a) positive and (b) negative ion mode. The proposed molecular structures for the most abundant peaks (1)-(3) are shown.

Although not detected in all plasma-treated tyrosine samples, 3-(4-hydroxyphenyl)-2-nitropropanoic acid was identified in the negative ion mode of the mass spectra, particularly in oxygen-treated conditions at 20min of plasma treatment (m/z 210.04 of Fig. S4).

Figure S4. a -b) Mass spectra of nitrate intermedia of the tyrosine; c) possible mechanism for Tyrosine mass fragmentation and nitrate intermedia produced.

Figure S5. Fragmentation pattern species 5 figure 3 (exact mass 228.0514) with fragmentation mechanism.

Figure S6. Fragmentation pattern species 8 figure 3 (exact mass 244.0463) with fragmentation mechanism.

Figure S7. Fragmentation pattern species 4 figure 4 (exact mass 241.0466) with fragmentation mechanism. loss

Chemical physical characterization

3.1 measure of pH

PH measurements were carried out by a Hanna Instruments™ HI8314-1 pH-meter within the first 30min of plasma processing. To prevent artifacts caused by liquid junction potential drift and reference electrode instability, induced by organic molecules and reactive species, glass electrode pH values were recorded within 3 seconds of immersion, yielding results consistent with litmus paper indicators and reported in figure S8.

Figure S8- pH measurements of water solutions of Cysteine (0.42 mM), Glucose (300mM), Tyrosine (1.65 mM), Resorcinol (2.72 mM) and double distilled water (ddH2O) before (0) and after 45s, 3min or 5min of plasma treatment fed with O_2_ or Air.
